# Supplementary figures and images for: SMRT long reads and Direct Label and Stain optical maps allow the generation of a high-quality genome assembly for the European barn swallow (Hirundo rustica rustica)
Source: Gigascience. 2018 Nov 29;8(1):giy142. doi: 10.1093/gigascience/giy142 (PMC6324554; doi:10.1093/gigascience/giy142)

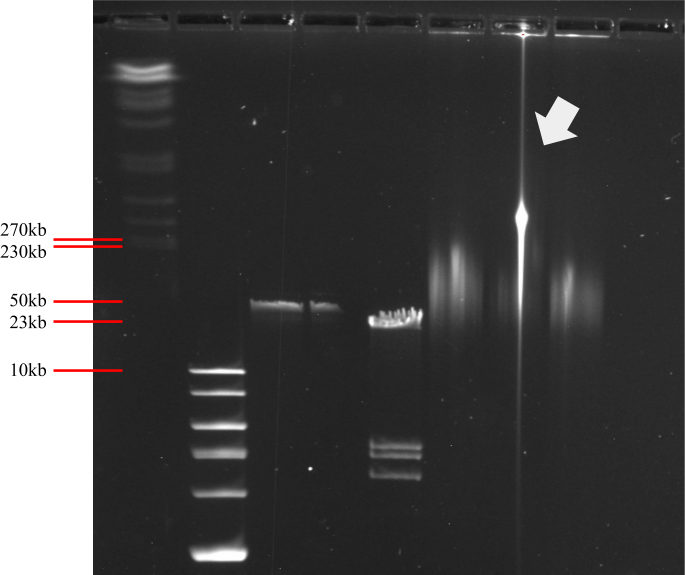

Supplement: giy142_Supplemental_Files [file giy142_supplemental_files.zip › Supplementary Figure 1.png]

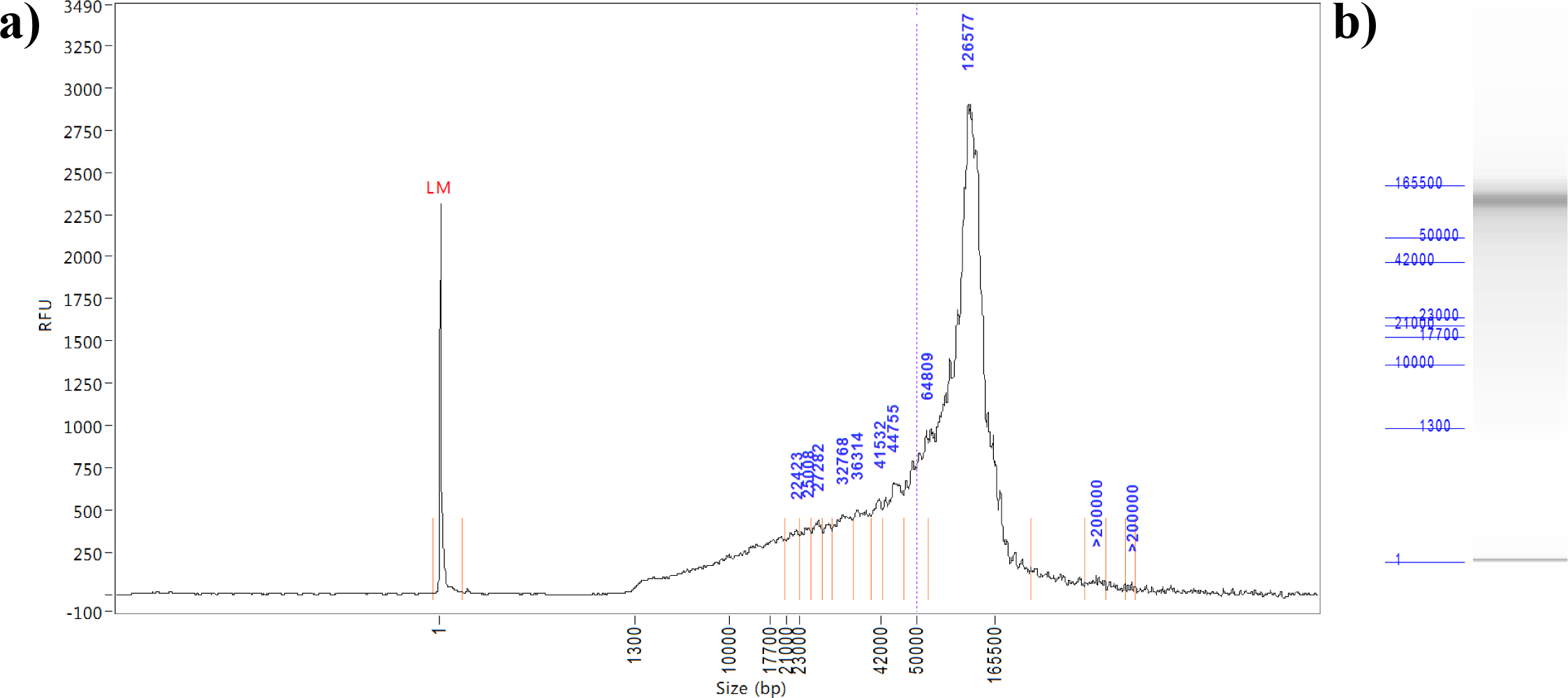

Supplement: giy142_Supplemental_Files [file giy142_supplemental_files.zip › Supplementary Figure 2.tif]

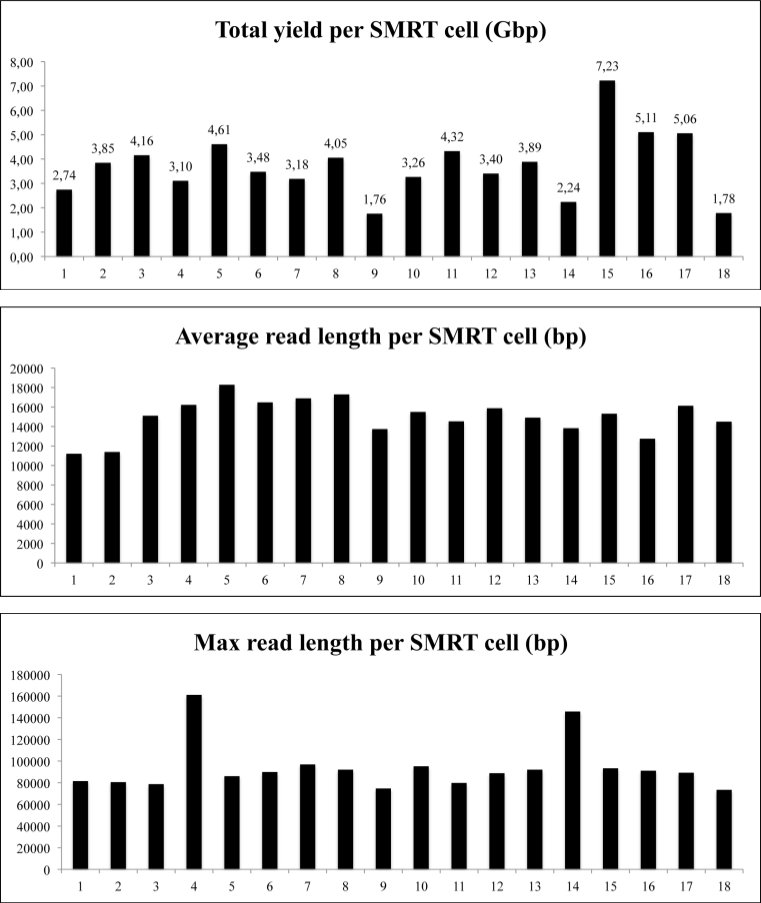

Supplement: giy142_Supplemental_Files [file giy142_supplemental_files.zip › Supplementary Figure 3.png]

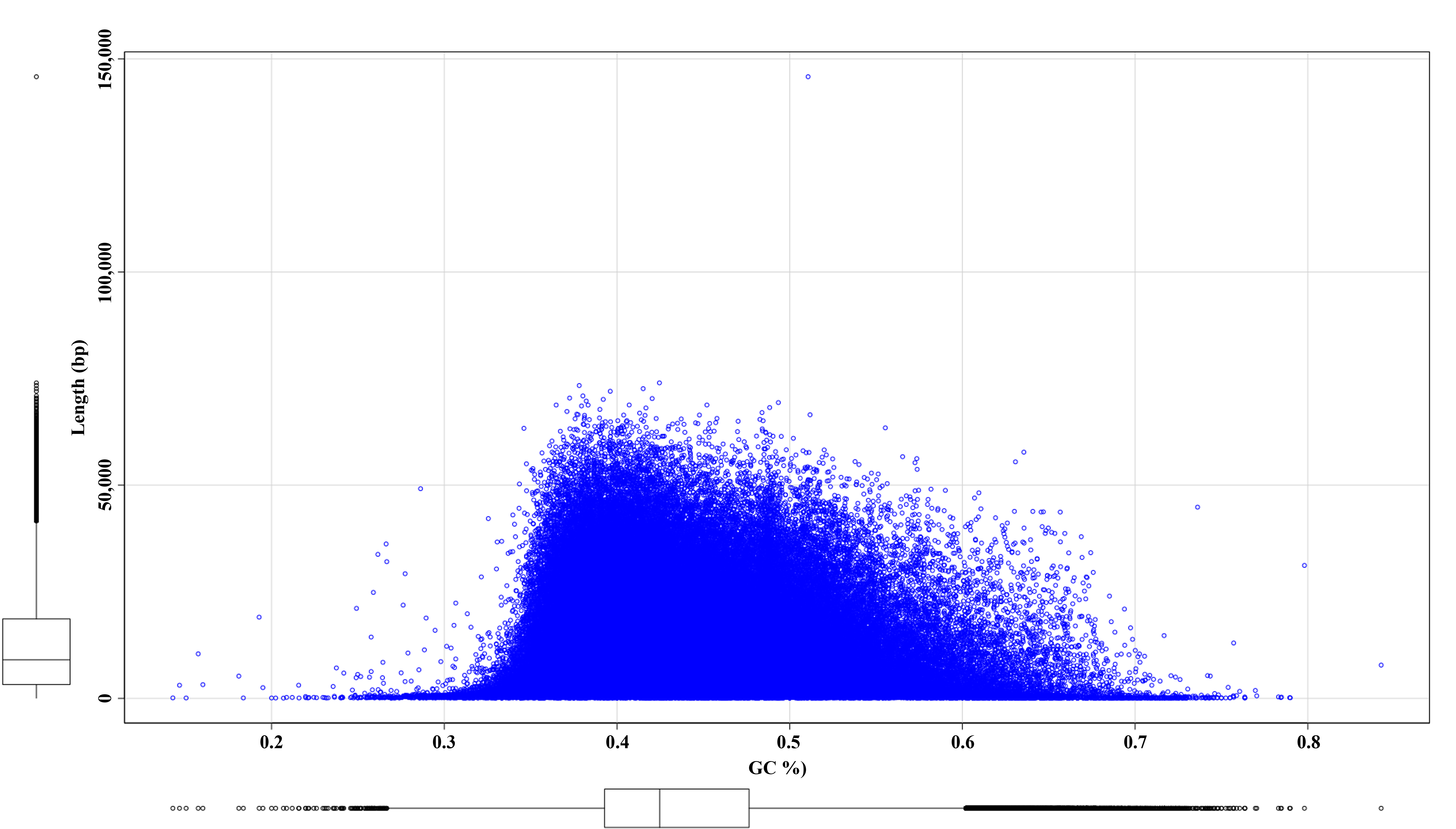

Supplement: giy142_Supplemental_Files [file giy142_supplemental_files.zip › Supplementary Figure 4.png]

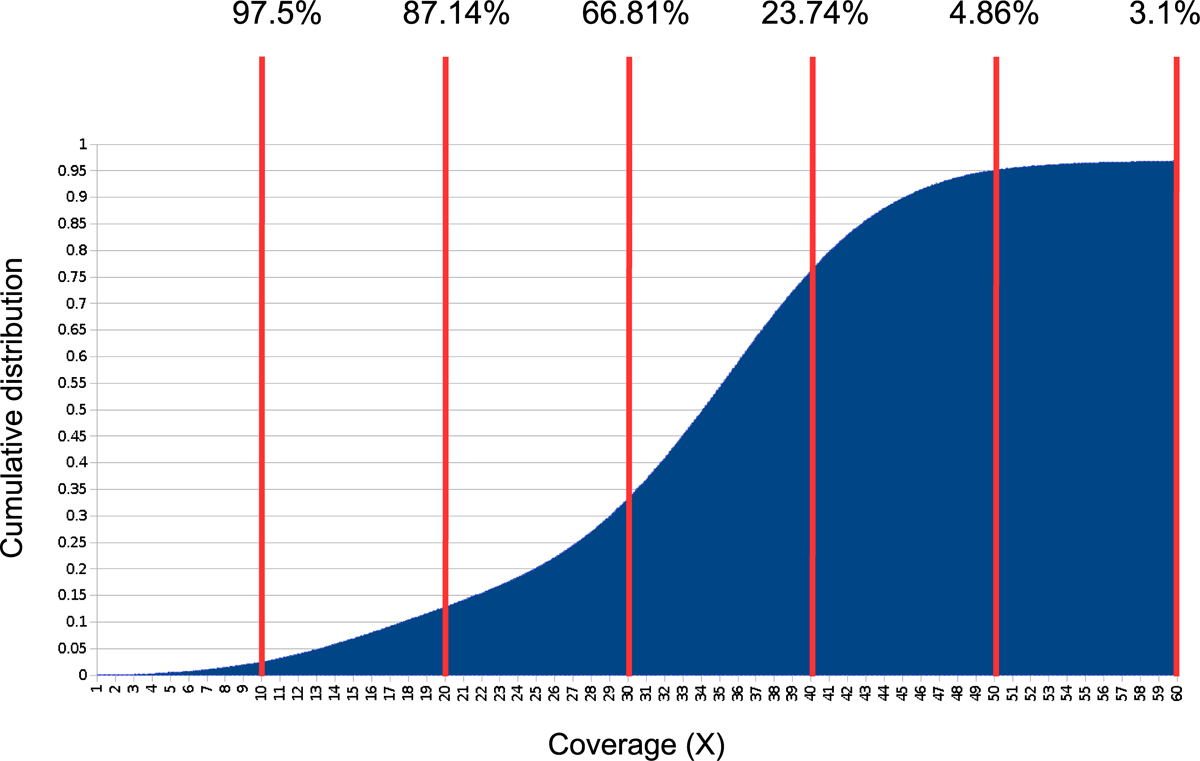

Supplement: giy142_Supplemental_Files [file giy142_supplemental_files.zip › Supplementary Figure 5.tif]
