# Supplementary material for: SMRT long reads and Direct Label and Stain optical maps allow the generation of a high-quality genome assembly for the European barn swallow (Hirundo rustica rustica)
Source: Gigascience. 2018 Nov 29;8(1):giy142. doi: 10.1093/gigascience/giy142 (PMC6324554; doi:10.1093/gigascience/giy142)
Supplement: giga-d-18-00272_original_submission.pdf [file giy142_giga-d-18-00272_original_submission.pdf]

GigaScience

SMRT long-read sequencing and Direct Label and Stain optical maps allow the generation of a high-quality genome assembly for the European barn swallow (Hirundo rustica rustica)

--Manuscript Draft--

**Manuscript Number:**	GIGA-D-18-00272
**Full Title:**	SMRT long-read sequencing and Direct Label and Stain optical maps allow the generation of a high-quality genome assembly for the European barn swallow (Hirundo rustica rustica)
**Article Type:**	Data Note
**Funding Information:**	California State Polytechnic University, Pomona — Dr. Andrea Bonisoli-Alquati
**Abstract:**	Background:<br>The barn swallow (Hirundo rustica) is a migratory bird that has been the focus of a large number of ecological, behavioural and genetic studies. To facilitate further population genetics and genomic studies, here we present a high-quality genome for the European subspecies (Hirundo rustica rustica).<br><br>Findings:<br>We have assembled a highly contiguous genome sequence using Single Molecule Real-Time (SMRT) DNA sequencing and Bionano optical maps. We compared and integrated optical maps derived both from the Nick, Label, Repair and Stain and from Direct Label and Stain technologies. For our SMRT-only assembly, the direct labelling system more than doubled the assembly N50 with respect to the nickase system. The dual enzyme hybrid scaffold led to a further marginal increase in scaffold N50 and an overall increase of confidence in scaffolds. After removal of haplotigs, the final assembly is approximately 1.21 Gbp in size, with an N50 value of over 25.95 Mbp, representing an improvement in N50 of over 650 fold with respect to a previously reported assembly based on paired-end short read data.<br><br>Conclusions:<br>This high-quality genome assembly represents a valuable resource for further studies of population genetics of the barn swallow and for studies concerning the evolution of avian genomes. It also represents the first genome assembled combining SMRT sequencing with the new Bionano Direct Label and Stain technology for scaffolding, highlighting the potential of this methodology to contribute to substantial increases in the contiguity of genome assemblies.
**Corresponding Author:**	Giulio Formenti<br>University of Milan<br>Milano, Mi ITALY
**Corresponding Author Secondary Information:**	
**Corresponding Author's Institution:**	University of Milan
**Corresponding Author's Secondary Institution:**	
**First Author:**	Giulio Formenti, Graduate student
**First Author Secondary Information:**	
**Order of Authors:**	Giulio Formenti, Graduate student<br>Matteo Chiara, Ph.D.<br>Lucy Poveda, Ph.D.<br>Kees-Jan Francoijs<br>Andrea Bonisoli-Alquati, Assistant Professor

Powered by Editorial Manager® and ProduXion Manager® from Aries Systems Corporation

	Luca Canova
	Luca Gianfranceschi, Associate Professor
	David Stephen Horner, Associate Professor
	Nicola Saino, Full Professor
**Order of Authors Secondary Information:**	
**Additional Information:**	
**Question**	**Response**
Are you submitting this manuscript to a special series or article collection?	No
**Experimental design and statistics**<br><br>Full details of the experimental design and statistical methods used should be given in the Methods section, as detailed in our Minimum Standards Reporting Checklist. Information essential to interpreting the data presented should be made available in the figure legends.<br><br>Have you included all the information requested in your manuscript?	Yes
**Resources**<br><br>A description of all resources used, including antibodies, cell lines, animals and software tools, with enough information to allow them to be uniquely identified, should be included in the Methods section. Authors are strongly encouraged to cite Research Resource Identifiers (RRIDs) for antibodies, model organisms and tools, where possible.<br><br>Have you included the information requested as detailed in our Minimum Standards Reporting Checklist?	Yes
**Availability of data and materials**<br><br>All datasets and code on which the conclusions of the paper rely must be either included in your submission or	Yes

deposited in publicly available repositories (where available and ethically appropriate), referencing such data using a unique identifier in the references and in the "Availability of Data and Materials" section of your manuscript.<br><br>Have you have met the above requirement as detailed in our Minimum Standards Reporting Checklist?	

Powered by Editorial Manager® and ProduXion Manager® from Aries Systems Corporation

Manuscript

Click here to download Manuscript Manuscript for submission.docx

SMRT long-read sequencing and Direct Label and Stain optical maps allow the generation of a high-quality genome assembly for the European barn swallow (*Hirundo rustica rustica*)

Giulio Formenti* (giulio.formenti@unimi.it), Department of Environmental Science and Policy, University of Milan (Milan, Italy).

Matteo Chiara* (matteo.chiara@unimi.it), Department of Biosciences, University of Milan (Milan, Italy).

Lucy Poveda (lucy.poveda@fgcz.uzh.ch), Functional Genomics Center of Zurich, University of Zurich, (Zurich, Switzerland).

Kees-Jan Francoijs (kfrancoijs@bionanogenomics.com), Bionano Genomics (San Diego, CA, USA).

Andrea Bonisoli-Alquati (aalquati@cpp.edu), Department of Biological Sciences, California State Polytechnic University (Pomona, CA, USA).

Luca Canova (canova@unipv.it), Department of Biochemistry, University of Pavia (Pavia, Italy).

Luca Gianfranceschi (luca.gianfranceschi@unimi.it), Department of Biosciences, University of Milan (Milan, Italy).

David Stephen Horner (david.horner@unimi.it), Department of Biosciences, University of Milan (Milan, Italy).

Nicola Saino (nicola.saino@unimi.it), Department of Environmental Science and Policy, University of Milan (Milan, Italy).

*These authors contributed equally to the work.

ABSTRACT (max 250 words)

Background:

The barn swallow (*Hirundo rustica*) is a migratory bird that has been the focus of a large number of ecological, behavioural and genetic studies. To facilitate further population genetics and genomic studies, here we present a high-quality genome for the European subspecies (*Hirundo rustica rustica*).

Findings:

We have assembled a highly contiguous genome sequence using Single Molecule Real-Time (SMRT) DNA sequencing and Bionano optical maps. We compared and integrated optical maps derived both from the Nick, Label, Repair and Stain and from Direct Label and Stain technologies. For our SMRT-only assembly, the direct labelling system more than doubled the assembly N50 with respect to the nickase system. The dual enzyme hybrid scaffold led to a further marginal increase in scaffold N50 and an overall increase of confidence in scaffolds. After removal of haplotigs, the final assembly is approximately 1.21 Gbp in size, with an N50 value of over 25.95 Mbp, representing an improvement in N50 of over 650 fold with respect to a previously reported assembly based on paired-end short read data.

Conclusions:

This high-quality genome assembly represents a valuable resource for further studies of population genetics of the barn swallow and for studies concerning the evolution of avian genomes. It also represents the first genome assembled combining SMRT sequencing with the new Bionano Direct Label and Stain technology for scaffolding, highlighting the potential of this methodology to contribute to substantial increases in the contiguity of genome assemblies.

Keywords: genome, barn swallow, third-generation sequencing, SMRT, long reads, Bionano, DLS, DLE-1, optical maps, single molecule.

Data Description

Context

The barn swallow is a passerine bird with at least 8 recognized subspecies in Europe, Asia and North America. The European barn swallow (*Hirundo rustica rustica*) (Figure 1) breeds in a broad latitudinal range, between 63-68°N and 20-30°N [1]. Numerous evolutionary and ecological studies have focussed on its biology, life history, sexual selection, and response to climate change. More recently, the barn swallow has become the focus of genetic studies on the divergence between subspecies [2–4] and on the control of phenological traits [5–8]. Due to its synanthropic habits and its cultural value, the barn swallow is also a flagship species in conservation biology [1]. The availability of high-quality genomic resources, including a reference genome, is thus pivotal to further boost the study and conservation of this species.

Figure 1: the European barn swallow (*Hirundo rustica rustica*). Courtesy of Chiara Scandolara.

In 2016, Safran and coworkers reported the first draft of the genome for the American subspecies (*Hirundo rustica erythrogaster*) constructed from Illumina paired-end reads at 47x coverage depth [2]. It has not been possible to analyze this assembly as neither the raw nor the assembled data were made publicly available. However, this assembly was described as containing 1.1 Gbp of assembled sequences (average contig length 11 kbp, contig N50 = 39 kbp, contig N90 = 3.8 kbp, longest scaffold: 732 kbp), compared to an estimated genome size of 1.28 Gbp [9]. Moreover, the assembly was derived from a male individual, excluding information for the W chromosome, as females are the heterogametic (ZW) sex in birds.

To address the aforementioned limitations, we have employed two single-molecule technologies, SMRT Third-Generation Sequencing (TGS) from Pacific Biosciences (Menlo Park, California, USA) and optical mapping from Bionano Genomics (San Diego, California, USA), to produce a state-of-the-art high-quality genome assembly for the European subspecies. For optical mapping we have labeled DNA molecules both with one of the original Nick, Label, Repair and Stain (NLRS) nickases

(Nb.BssSI) and with the new Direct Label and Stain (DLS) approach (enzyme DLE-1). The latter technique was officially released in February 2018 and avoids nicking and subsequent cleavage of DNA molecules during staining [10]. We show that, at least with our data, DLS allows a considerable improvement of scaffold contiguity with respect to the nickase tested. Furthermore, the “dual enzyme” approach affords additional support for scaffold junctions. To our knowledge this genome assembly is the first to incorporate DLS data, and their integration with SMRT sequencing provided assembly contiguity metrics well in excess of those specified for “Platinum genomes” by the Vertebrate Genomes Project (VGP) [11].

Blood sample collection

The blood used as a source of DNA was derived from a minimally invasive sampling performed on a female individual of approximately two years of age during May 2017 in a farm near Milan in Northern-Italy (45.4N 9.3E). Blood was collected in heparinized capillary tubes. Three hours after collection, the sample was centrifuged to separate blood cells from plasma and then stored at -80°C.

DNA extraction and quality control for SMRT library preparation

DNA extraction was performed on blood cells portion of centrifuged whole blood containing nucleated erythrocytes and leukocytes using the Wizard genomic DNA purification kit (Promega, Cat. No. A1125). This kit employs a protocol similar to classical Phenol/Chloroform DNA extraction, with no vortexing steps after cell lysis. After purification, DNA quality and concentration was assessed by Nanodrop (Thermo Fisher Scientific, Cat. No. ND-1000) and subsequently by Pulsed Field Gel Electrophoresis (PFGE). Detectable DNA was over 23 kbp in size, with the vast majority over 50 kbp and even over 200 kbp (Supplementary Figure 1). PFGE quality results were further confirmed by capillary electrophoresis on FEMTO Pulse instrument (AATI, Cat. No. FP-1002-0275) (Supplementary Figure 2). DNA was stored at -80°C and shipped on dry ice.

SMRT library preparation and sequencing

SMRTbell Express Template Prep Kit (Pacific Biosciences, Cat. No. 101- 357- 000) was used to produce the insert library. Input gDNA concentration was measured on a Qubit Fluorometer dsDNA Broad Range (Life Technologies, Cat. No. 32850). 10µg of gDNA was mechanically sheared to an average size distribution of 40-50 kbp, using a Megaruptor Device (Diagenode, Cat. No. B06010001). FEMTO Pulse capillary electrophoresis was employed to assess the size of the fragments. 5 µg of sheared gDNA was DNA-damage repaired and end-repaired using polishing enzymes. Blunt-end ligation was used to create the SMRTbell template. A Blue Pippin device (Sage Science, Cat. No. BLU0001) was used to size-select the SMRTbell template and enrich for fragments > 30 kbp, excluding the first two cells for which the library was enriched for fragments > 15 kbp. The size-selected library was checked using FEMTO Pulse and quantified on a Qubit Fluorometer. A ready to sequence SMRT bell-Polymerase Complex was created using the Sequel binding kit 2.0 (Pacific Biosciences, Cat. No. 100- 862- 200). The Pacific Biosciences Sequel instrument was programmed to sequence the library on 18 Sequel SMRT Cells 1M v2 (Pacific Biosciences, Cat. No. 101- 008-000), taking one movie of 10 hours per cell, using the Sequel Sequencing Kit 2.1 (Pacific Biosciences, Cat. No. 101- 310- 400). After the run, sequencing data quality was checked via the PacBio SMRT Link v5.0.1 software using the “run QC module”. An average of 3.7 Gbp (standard deviation: 1.7) were produced per SMRT cell (average N50 read length = 25,622 bp), with considerable improvements between the 15 kbp library and the 30 kbp library (see Supplementary Figure 3 for more detailed statistics). We observed a wide distribution in the GC content of reads (Supplementary Figure 4). This is likely explained by the presence in avian genomes of three classes of chromosomes: macrochromosomes (50-200 Mbp, 5 in chicken), intermediate chromosomes (20-40 Mbp, 5 in chicken) and microchromosomes (12 Mbp on average, 28 in chicken). These last account for only 18% of the total genome but harbor ~31% of all chicken genes, have higher recombination rates and higher GC contents on average [12].

Assembly of SMRT reads

The final assembly of long reads was conducted with software CANU v1.7 [13] using default parameters except for the “correctedErrorRate” which was set at 0.075. The assembly processes occupied 3,840 CPU hours and 2.2 Tb of RAM for read correction, 768 CPU hours and 1.1 Tb of RAM for the trimming steps, and 3280 CPU hours and 2.2 Tb of RAM for the assembly phase. The assembly contained 3,872 contigs with a N50 of 5,2 Mbp for a total length of the assembly of 1311.7 Mbp (Table 1 and Supplementary Table 1). Final polishing was performed using the Arrow v2.10 software (Pacific Biosciences) and resulted in final coverage of 45.4x.

Cell count and DNA extraction for optical mapping

High-molecular weight (HMW) DNA was extracted from 7-8 µl of the cell portion from the same blood sample used for SMRT sequencing using the Blood and Cell Culture DNA Isolation kit (Bionano Genomics, Cat. No. RE-016-10). HMW DNA was extracted by embedding cells in low melting temperature agarose plugs that were incubated with Proteinase K (Qiagen, Cat. No. 158920) and RNAseA (Qiagen, Cat. No. 158924). The plugs were washed and solubilized using Agarase Enzyme (Thermo Fisher Scientific, Cat. No. EO0461) to release HMW DNA and further purified by drop dialysis. DNA was homogenised overnight prior to quantification using a Qubit Fluorometer.

***In silico* digestion**

The genome assembly obtained with CANU was *in silico* digested using Bionano Access software to test whether the nicking enzyme (Nb.BssSI), with recognition sequence (CACGAG), and the non-nicking enzyme DLE-1, with recognition sequence (CTTAAG), were suitable for optical mapping in our bird genome. An average of 16.9 nicks/100 kbp with a nick-to-nick distance N50 of 11,708 bp were expected for Nb.BssSI, while DLE-1 was found to induce 19.1 nicks/100 kbp with a nick-to-nick distance N50 of 8,775 bp, both in line with manufacturer’s requirements.

DNA labeling for optical mapping

For NLRS, DNA was labeled according to manufacturer’s instructions using the Prep DNA Labeling Kit-NLRS (Bionano Genomics, Cat. No. 80001). 300 ng of purified genomic DNA was nicked with

Nb.BssSI (New England Biolabs, Cat. No. R0681S) in NEB Buffer 3. The nicked DNA was labeled with a fluorescent-dUTP nucleotide analog using Taq DNA polymerase (New England BioLabs, Cat. No. M0267S). After labeling, nicks were ligated with Taq DNA ligase (New England BioLabs, Cat. No. M0208S) in the presence of dNTPs. The backbone of fluorescently labeled DNA was counterstained overnight with YOYO-1 (Bionano Genomics, Cat. No. 80001).

For DLS, DNA was labeled using the Bionano Prep DNA Labeling Kit-DLS (Cat. No. 80005) according to manufacturer's instructions. 750 ng of purified genomic DNA was labeled with DLE labeling Mix and subsequently incubated with Proteinase K (Qiagen, Cat. No. 158920) followed by drop dialysis. After the clean-up step, the DNA was pre-stained, homogenised and quantified using on a Qubit Fluorometer to establish the appropriate amount of backbone stain. The reaction was incubated at room temperature for at least 2 hours.

Generation of optical maps

NLRS and DLS labeled DNA were loaded into a nanochannel array of a Saphyr Chip (Bionano Genomics, Cat. No. FC-030-01) and run by electrophoresis each into a compartment. Linearized DNA molecules were imaged using the Saphyr system and associated software (Bionano Genomics, Cat. No. 90001 and CR-002-01). In the experiment with DLE-1, molecule N50 was 0.2475 Mbp for molecules above 20 kbp and 0.3641 Mbp for molecules above 150 kbp - with an average label density of 15.7/100 kbp for molecules above 150 kbp. Map rate was 56.4% for molecules above 150 kbp. Effective coverage was 30.6x.

In the experiment with Nb.BssSI, molecule N50 was 0.1298 Mbp for molecules above 20 kbp and 0.2336 Mbp for molecules above 150 kbp - with an average label density of 11.8/100 kbp for molecules above 150 kbp. Map rate was 38.9% for molecules above 150 kbp. Effective coverage was 28.2x. Using both DLE-1 and Nb.BssSI, label metrics were in line with the manufacturer's expectations.

Assembly of optical maps

The *de novo* assembly of the optical maps was performed using the Bionano Access v1.2.1 and Bionano Solve v3.2.1 software. The assembly type performed was the "non-haplotype" with "no extend split" and "no cut segdups". Default parameters were adjusted to accommodate the genomic properties of the barn swallow genome. Specifically, given the size of the genome, the minimal length for the molecules to be used in the assembly was reduced to 100 kbp, the "Initial P-value" cut off threshold was adjusted to 1×10^{-10} and the P-value cut off threshold for extension and refinement was set to 1×10^{-11} according to manufacturer's guidelines (default values are 150 kbp, 1×10^{-11} and 1×10^{-12} respectively).

A total of 233,450 (of 530,527) NLRS-labelled molecules (N50 = 0.2012 Mbp) were aligned to produce 2,384 map fragments with an N50 of 0.66 Mbp for a total length of 1338.6 Mbp (coverage = 32x). 108,307 (of 229,267) DLE-1 labelled input DNA molecules with a N50 of 0.3228 Mbp (theoretical coverage of the reference 48x) produced 555 maps with a N50 length of 12.1 Mbp for a total length 1299.3 Mbp (coverage = 23x).

Hybrid scaffolding

Single and dual enzyme Hybrid Scaffolding (HS) was performed using Bionano Access v1.2.1 and Bionano Solve v3.2.1. For the dual enzyme and DLE-1 scaffolding, default settings were used to perform the HS. For Nb.BssSI the "aggressive" settings were used without modification. The NLRS hybrid assembly had an N50 of 8.3 Mbp (scaffold only N50 = 10.8 Mbp) for a total length of 1,338.6 Mbp (total length of scaffolded contigs = 1,175.3 Mbp) and consisted of 409 scaffolds and 2,899 un-scaffolded contigs. The DLS hybrid assembly had N50 of 17.3 Mbp (scaffold only N50 = 25.9 Mbp) for a total length of 1,340.2 Mbp (total length of scaffolded contigs = 1,148.4 Mbp) and consisted of 211 scaffolds and 3,106 un-scaffolded contigs. Dual enzyme HS (incorporating both DLS and NLRS maps) resulted in an assembly with N50 of 23.8 Mbp (scaffold only N50 = 28.4 Mbp) for a total length of 1,351.8 Mbp (total length of scaffolded contigs = 1,208.8 Mbp) and consisted of 273 scaffolds and 2,810 un-scaffolded contigs. During the automatic conflict resolution in the dual enzyme HS, 185 SMRT contigs were cut, as Bionano maps confidently indicated mis-assemblies of the SMRT reads. Conversely 117 bionano maps were cut indicating that the chimeric score did not

provide sufficient confidence to cut the assembly based on SMRT contigs. Of 3,872 SMRT contigs, 1,243 (32%) were anchored in the Bionano maps (of which 990 were anchored in both DLS and NLRS maps). 56 and 226 were anchored in DLS and NLRS maps respectively. 2810 maps could not be anchored at all.

Notably, all hybrid assemblies were somewhat larger than the expected genome size, and in all cases, the N50 of un-scaffolded contigs was extremely low (0.06 Mbp for the dual enzyme hybrid assembly). We hypothesized that a significant proportion of these small contigs might represent divergent homologous haplotigs that were assembled independently. Similarity searches were consistent with this possibility as almost 95% of the contigs that were not scaffolded in the dual enzyme hybrid assembly showed > 98% identity to scaffolded contigs over 75% of their length or more. These contigs were discarded, resulting in a final assembly (Table 1 and Supplementary Table 1 for detailed statistics) of 1.21 Gbp (N50 = 25.9 Mbp) made up of 273 dual enzyme hybrid scaffolds (N50 = 28.42 Mbp) and 91 un-scaffolded contigs (N50 = 0.0644 Mbp). The final assembly is slightly smaller than the previously estimated genome size (1.28 Gbp) [9], possibly reflecting an imprecise older estimate and/or the possibility that some poorly assembled repeats were discarded in the final step described above. The average read SMRT read coverage for the genome assembly was 34.15X (implying a theoretical QV of over 40). Supplementary Figure 5 provides a summary of observed sequence coverage depth.

	**Safran et al.** [2][1]	**SMRT contigs**[2]	**Final assembly**[3]
Species	*H. r. erythrogaster*	*H. r. rustica*	
Starting raw data (Gbp)	61.7	66.4	59.6
N50 (bp)	38844	5189284	25954216
N90 (bp)	3718	85340	2002624
Total size (Gbp)	1.1	1.31	1.21
Theoretical genome coverage*	47x	52x	47x
% genome coverage*	85.9	102.6	94.5
# of contigs/scaffolds	100153	3872	364

Avg contig/scaffold length (bp)	11010	338782	3334461
Longest contig/scaffold (bp)	732517	33230000	98053015

Table 1: Assembly metrics for contigs and final scaffolds in our European barn swallow genome compared to the published American barn swallow genome. [1] Illumina PE reads assembled using SOAPdenovo v2.04 [14]. [2] SMRT reads assembled using CANU v1.7 [13]. [3] SMRT contigs assembled with CANU and scaffolded using Bionano dual enzyme HS, with haplotigs removed as detailed in the text. *Based on a barn swallow genome size estimate of 1.28 Gbp [9].

Annotation of genes and repeats

With respect to mammals, avian genomes generally contain relatively low proportions of repetitive sequences and show strong mutual synteny [15]. This appears to be the case also for the barn swallow genome. In particular, 7.11% of the final assembly was annotated as repetitive using RepeatMasker [16], with the major contributions deriving from L2/CR1/Rex LINE elements (3.37%), retroviral LTRs (1.59%) and simple repeats (1.56%). These repeats were soft-masked prior to *de novo* gene prediction using Augustus [17] with *Gallus gallus* gene models.

In all, 35,644 protein coding genes were predicted, of which 9,189 were overlapped by more than 30% of their size with repetitive genomic elements. Of the remaining 26,455 predicted protein coding genes, 24,331 harbored a PFAM protein domain. Simple similarity searches based on blastp [18] (with default parameters) suggested that 17,895 of the predicted protein coding genes have a best reciprocal blast hit with gene models derived from *Gallus gallus* GRCg6a assembly (as available from [19]), while 2,927 of the proteins predicted by Augustus did not show any significant match (e-value $<= 1 \times 10^{-15}$, identity > 35%).

BUSCO genes

Of a total of 4915 conserved bird Benchmarking with Universal Single-Copy Orthologs (BUSCO) groups [20] sought, 4,598 (93.6%) were complete, 4,521 (92.0%) were complete and single-copy, 77 (1.6%) were complete and duplicated, 192 (3.9%) were fragmented and 125 (2.5%) were missing. The

percentage of contiguously assembled BUSCO genes is consistent with recent results with Anna's Hummingbird (*Calypte anna*) and the Zebra Finch (*Taeniopygia guttata*) [21]. We note that 40 of the "missing" bird BUSCO genes are absent from at least 2 of the 54 available avian genome sequences, suggesting that, despite the potentially incomplete nature of some draft genomes, some of these genes may not be universally conserved among birds.

Synteny with the Chicken and Hummingbird genomes

Alignment of the final assembly with the most recent assembly of the chicken genome (GRCg6a) using D-Genies [22] indicates high levels of collinearity between these two genomes with a limited number of intra-chromosomal rearrangements (Figure 2). The high level of collinearity between independently assembled and scaffolded sequences provides circumstantial support for the quality of both the contigs and the hybrid scaffolds and is consistent with previous observations of high levels of synteny and minimal inter-chromosomal rearrangements among birds [15].

Conclusion

During the last 20 years, nucleic acid sequencing technologies have developed over four times faster than improvements of microchip complexity predicted by Moore's law [23,24]. While short-read NGS (now known as Second Generation Sequencing - SGS) technologies have allowed the production of cost-effective genome drafts for many birds and other vertebrate species [25–27], the reduction in genome sequencing costs has typically come at the price of compromises in contiguity and accuracy of assemblies with respect to earlier efforts based on Sanger reads and extensive physical mapping [28]. Many limitations of SGS-based assemblies stem from the occurrence of long sequence repeats. In many animal species, transposons are frequently located in introns [29] and the presence of large gene families of closely related paralogs can lead to the existence of long "genic" repeats. Accordingly, even apparently contiguous genic regions can feature juxtaposition of paralogous gene fragments [21]. Given the inception of large scale sequencing initiatives aiming to produce genome assemblies for a wide range of organisms [30–33], it is critical to identify combinations of sequencing and scaffolding approaches that allow the cost effective generation of

genuinely high-quality genome assemblies [11]. While exhibiting higher rates of single-base errors than some SGS approaches, TGS technologies, including SMRT sequencing, offer read-lengths unparalleled by SGS or Sanger sequencing [34]. Moreover, recent and ongoing improvements in TGS methods are rapidly reducing the "per-base" cost of TGS data compared to that of SGS. On the other hand, as an alternative to scaffolding with long insert mate-pairs [35] or to chromatin proximity ligation sequencing [36], contiguity and accuracy of long-read-based assemblies can be further improved by optical mapping. This relies on nanoscale channels that can accommodate thousands of single, ultralong (>200 kbp) double-stranded DNA filaments in parallel, subsequently stained to recognize specific 8-9 bp long motifs [37]. The combination of long reads and optical maps has already proven invaluable to produce high-quality genome assemblies, even in the case of particularly complex genomes [38]. Here, using only SMRT sequencing and Bionano optical maps we have produced a high-quality and contiguous genome for the barn swallow. With respect to a previously reported SGS-based assembly of the American barn swallow genome using a comparable amount of raw data [2], even the contigs generated from long-read sequencing alone show an 134-fold increase in N50. In terms of N50, number and average length, these contigs are similar to those recently obtained for the Anna's hummingbird genome using the same technology [21]. Furthermore, the fold change in N50 attained by Bionano NLRS hybrid scaffolding of the European barn swallow genome (1.6 fold before removal of haplotigs) is comparable with results obtained by other genome assemblies that have employed this method [39]. Strikingly, the new DLS method greatly outperformed the NLRS system, providing a 3.3 fold increase of N50 (before removal of haplotigs). Moreover, incorporation of both labelling systems into the hybrid scaffolding yielded a final assembly showing 5-fold improvement of the N50 with respect to the original SMRT assembly, simultaneously providing "independent" validation of many scaffold junctions. We note that the presence of numerous microchromosomes in avian genomes restricts the final N50 value potentially attainable for the assembly, as the fully assembled karyotype would have an N50 of ~ 90 Mbp. Yet, after removal of putative haplotigs, our genome assembly contiguity metrics meet the high standards of the VGP consortium "Platinum Genome" criteria (contig N50 in excess of 1 Mbp and scaffold N50 above 10 Mbp) [11]. Accordingly, we believe that the data presented here, as well as attesting to the

effectiveness of SMRT sequencing combined with DLS optical mapping for the assembly of vertebrate genomes, will provide an invaluable asset for population genetics studies in the barn swallow and for comparative genomics in birds.

Re-use Potential

Future directions for the barn swallow genome include the phasing of the assembly to generate extended haplotypes, a more thorough gene annotation using RNA/IsoSeq sequencing data, detailed comparisons with genome data from *Hirundo rustica erythrogaster,* re-evaluation of data from previous population genetics studies conducted in this species, as well as characterization of the epigenetic landscape.

Availability of supporting data

The data sets supporting the results of this article are available in the GenBank repository, under Bioproject PRJNA481100.

Competing interests

Kees-Jan Francoijs is currently employed at Bionano Genomics (San Diego, CA, USA). All other authors declare no competing interest.

Funding

Funding to A.B.-A. was provided by Cal Poly Pomona College of Science.

Authors' contributions

G.F, N.S., A.B.-A., L.G., D.S.H, M.C. and L.C. conceived the project and designed the experiments; G.F. performed DNA extraction and quality control; M.C. carried out CANU assemblies, gene and repeat annotation. D.S.H., M.C. and L.G. performed other bioinformatics analyses; L.P. conducted the optical mapping; K.J.F. produced the hybrid scaffolds; G.F., D.S.H, M.C., N.S. and L.C. drafted the manuscript. All authors edited and contributed to the manuscript.

Acknowledgements

We thank Manuela Caprioli for support in field work, sample collection, DNA extraction and quality control as well as Dr. Elena Galati for support in PFGE quality control. We also thank The Functional Genomics Center of Zurich, where SMRT sequencing and optical mapping were carried out, and particularly Andrea Patrignani for SMRT sequencing. We thank Chiara Scandolara for the barn swallow picture used for Figure 1. We acknowledge the support of ELIXIR-IT and CINECA (HPC@CINECA) for provision of computational resources for SMRT read assembly.

Ethics approval

The blood sample used to generate the genomic data derived from a minimally invasive sampling on a single individual. Appropriate consent was obtained from the local authorities (Regione Lombardia).

Additional files

Supplementary Figure 1 (Supplementary Figure 1.png)

PFGE on a 1x agarose gel run for 18 hours at 160 mV. The two lowest overlapping bands in lane 1 represent yeast chromosomes of 230 kbp and 270 kbp, respectively. Lane 2 contains 1kb DNA ladder (highest 10 kbp), lane 3 and 4 the undigested lambda phage (50 kbp) and lane 5 digested lambda (upper band 23 kbp). Lane 7 contains the sample used in the study.

Supplementary Figure 2 (Supplementary Figure 2.tif)

FEMTO Pulse capillary electrophoresis results for the DNA sample used in the study.

Supplementary Figure 3 (Supplementary Figure 3.png)

Summary statistics for each SMRT cell employed.

Supplementary Figure 4 (Supplementary Figure 4.png)

GC content distribution in all sequence reads.

Supplementary Figure 5 (Supplementary Figure 5.png)

Cumulative coverage distribution of the final (de-haplotyped) assembly of the barn swallow genome. Coverage is indicated on the X axis. Red lines are used to display the proportion of the genome covered by more than 10, 20, 30, 40, 50 or 60 reads respectively.

Supplementary Table 1 (Supplementary Table 1.xlsx)

Comparison of assembly metrics for contigs and scaffolds between different assemblies. In hybrid scaffolds, the first column refers to assemblies including the un-scaffolded contigs while the second column only includes scaffolded contigs metrics. The estimated genome size of 1.28 Gbp is from [9]. Average gene size was estimated according to the latest available annotation of the *Gallus gallus* genome (GRCg6a).

List of abbreviations

DLS, Direct Label and Stain; HMW, High Molecular Weight; HS, Hybrid Scaffold; NGS, Next Generation Sequencing; NLRS, Nick, Label, Repair and Stain; N50, the shortest sequence length at 50% of the genome; N90, the shortest sequence length at 90% of the genome; PFGE, Pulsed Field Gel Electrophoresis; QV, Quality Value; SGS, Second Generation Sequencing; SMRT, Single Molecule Real-Time; TGS, Third Generation Sequencing; VGP, Vertebrate Genomes Project.

References

1. Turner A. The barn swallow. T & AD Poyser, London; 2006.

2. Safran RJ, Scordato ESC, Wilkins MR, Hubbard JK, Jenkins BR, Albrecht T, et al. Genome-wide differentiation in closely related populations: the roles of selection and geographic isolation. Mol Ecol. 2016;25:3865–83.

3. von Rönn JAC, Shafer ABA, Wolf JBW. Disruptive selection without genome-wide evolution across a migratory divide. Mol Ecol. 2016;25:2529–41.

4. Scordato ESC, Wilkins MR, Semenov G, Rubtsov AS, Kane NC, Safran RJ. Genomic variation across two barn swallow hybrid zones reveals traits associated with divergence in sympatry and allopatry. Mol Ecol. 2017;26:5676–91.

5. Caprioli M, Ambrosini R, Boncoraglio G, Gatti E, Romano A, Romano M, et al. Clock gene variation is associated with breeding phenology and maybe under directional selection in the migratory barn swallow. PLoS One. 2012;7:e35140.

6. Saino N, Romano M, Caprioli M, Fasola M, Lardelli R, Micheloni P, et al. Timing of molt of barn swallows is delayed in a rare Clock genotype. PeerJ. 2013;1:e17.

7. Bazzi G, Ambrosini R, Caprioli M, Costanzo A, Liechti F, Gatti E, et al. Clock gene polymorphism and scheduling of migration: a geolocator study of the barn swallow Hirundo rustica. Sci Rep. 2015;5:12443.

8. Saino N, Ambrosini R, Albetti B, Caprioli M, De Giorgio B, Gatti E, et al. Migration phenology and breeding success are predicted by methylation of a photoperiodic gene in the barn swallow. Sci Rep. 2017;7:45412.

9. Andrews CB, Mackenzie SA, Gregory TR. Genome size and wing parameters in passerine birds. Proc Biol Sci. 2009;276:55–61.

10. DLS announcement by Bionano Genomics at AGBT [Internet]. Available from: https://bionanogenomics.com/wp-content/uploads/2018/02/Bionano-AGBT2018-DLS_launch_final.pdf

11. Lewin HA, Robinson GE, Kress WJ, Baker WJ, Coddington J, Crandall KA, et al. Earth BioGenome Project: Sequencing life for the future of life. Proc Natl Acad Sci U S A. 2018;115:4325–33.

12. Kadi F, Mouchiroud D, Sabeur G, Bernardi G. The compositional patterns of the avian genomes and their evolutionary implications. J Mol Evol. 1993;37:544–51.

13. Koren S, Walenz BP, Berlin K, Miller JR, Bergman NH, Phillippy AM. Canu: scalable and accurate long-read assembly via adaptive k-mer weighting and repeat separation. Genome Res. 2017;27:722–36.

14. Li R, Zhu H, Ruan J, Qian W, Fang X, Shi Z, et al. De novo assembly of human genomes with massively parallel short read sequencing. Genome Res. 2010;20:265–72.

15. Ellegren H. Evolutionary stasis: the stable chromosomes of birds. Trends Ecol Evol. 2010;25:283–91.

16. Smit AFA, Hubley R, Green P. RepeatMasker Open-3.0 [Internet]. 1996–2010. Available from:

http://www.repeatmasker.org

17. Stanke M, Steinkamp R, Waack S, Morgenstern B. AUGUSTUS: a web server for gene finding in eukaryotes. Nucleic Acids Res. 2004;32:W309–12.

18. Altschul SF, Gish W, Miller W, Myers EW, Lipman DJ. Basic local alignment search tool. J Mol Biol. 1990;215:403–10.

19. Gallus gallus Proteins [Internet]. NCBI. Available from: https://www.ncbi.nlm.nih.gov/genome/proteins/111?genome_assembly_id=374862

20. Simão FA, Waterhouse RM, Ioannidis P, Kriventseva EV, Zdobnov EM. BUSCO: assessing genome assembly and annotation completeness with single-copy orthologs. Bioinformatics. 2015;31:3210–2.

21. Korlach J, Gedman G, Kingan SB, Chin C-S, Howard JT, Audet J-N, et al. De novo PacBio long-read and phased avian genome assemblies correct and add to reference genes generated with intermediate and short reads. Gigascience. 2017;6:1–16.

22. Cabanettes F, Klopp C. D-GENIES: dot plot large genomes in an interactive, efficient and simple way. PeerJ. 2018;6:e4958.

23. Heather JM, Chain B. The sequence of sequencers: The history of sequencing DNA. Genomics. 2016;107:1–8.

24. Stein LD. The case for cloud computing in genome informatics. Genome Biol. 2010;11:207.

25. Genome 10K Community of Scientists. Genome 10K: a proposal to obtain whole-genome sequence for 10,000 vertebrate species. J Hered. 2009;100:659–74.

26. Zhang G, Li C, Li Q, Li B, Larkin DM, Lee C, et al. Comparative genomics reveals insights into avian genome evolution and adaptation. Science. 2014;346:1311–20.

27. Jarvis ED, Mirarab S, Aberer AJ, Li B, Houde P, Li C, et al. Whole-genome analyses resolve early branches in the tree of life of modern birds. Science. 2014;346:1320–31.

28. Henson J, Tischler G, Ning Z. Next-generation sequencing and large genome assemblies. Pharmacogenomics. 2012;13:901–15.

29. Sela N, Kim E, Ast G. The role of transposable elements in the evolution of non-mammalian vertebrates and invertebrates. Genome Biol. 2010;11:R59.

30. Koepfli K-P, Paten B, Genome 10K Community of Scientists, O'Brien SJ. The Genome 10K Project: a way forward. Annu Rev Anim Biosci. 2015;3:57–111.

31. Zhang G, Rahbek C, Graves GR, Lei F, Jarvis ED, Gilbert MTP. Genomics: Bird sequencing project takes off. Nature. 2015;522:34.

32. Pennisi E. Sequencing all life captivates biologists. Science. 2017;355:894–5.

33. Teeling EC, Vernes SC, Dávalos LM, Ray DA, Gilbert MTP, Myers E, et al. Bat Biology, Genomes, and the Bat1K Project: To Generate Chromosome-Level Genomes for All Living Bat Species. Annu Rev Anim Biosci. 2018;6:23–46.

34. Bleidorn C. Third generation sequencing: technology and its potential impact on evolutionary biodiversity research. System Biodivers. Taylor & Francis; 2016;14:1–8.

35. Hunt M, Newbold C, Berriman M, Otto TD. A comprehensive evaluation of assembly scaffolding tools. Genome Biol. 2014;15:R42.

36. Burton JN, Adey A, Patwardhan RP, Qiu R, Kitzman JO, Shendure J. Chromosome-scale scaffolding of de novo genome assemblies based on chromatin interactions. Nat Biotechnol. 2013;31:1119–25.

37. Lam ET, Hastie A, Lin C, Ehrlich D, Das SK, Austin MD, et al. Genome mapping on nanochannel arrays for structural variation analysis and sequence assembly. Nat Biotechnol. 2012;30:771–6.

38. Nowoshilow S, Schloissnig S, Fei J-F, Dahl A, Pang AWC, Pippel M, et al. The axolotl genome and the evolution of key tissue formation regulators. Nature. 2018;554:50–5.

39. Gao Y, Wang H, Liu C, Chu H, Dai D, Song S, et al. De novo genome assembly of the red silk cotton tree (Bombax ceiba). Gigascience [Internet]. 2018;7. Available from: http://dx.doi.org/10.1093/gigascience/giy051

Click here to download Table Supplementary Table 1.xlsx

Comparison of barn swallow genome assemblies

	Safran	SMRT contigs	SMRT vs Saf[illegible]
**Strategy**	**Illumina 101 PE**	**PacBio Sequel**	**Fold-change**
**Starting raw data (Gbp)**	61.7	66.4	
**N50 (Mbp)**	0.039	5.189	134
**N90 (Mbp)**	0.0037180	0.093	25
**Total size (Gbp)**	1.1	1.312	1.2
**Theroretical genome coverage***	47	51.88	[illegible]
**% genome coverage***	85.9	102.5	1.2
**# of contigs/scaffolds**	100153	3872	26
**Avg contig/scaffold length (Mbp)**	0.011	0.339	31
**Longest contig/scaffold (Mbp)**	0.733	33.230	45
**Expected # of genes per contig¤**	1	26	31

* Barn swallow estimated genome size: 1.28 Gbp (Andrews et al. 2009)

¤ Estimate based on average protein coding gene size for latest available annotation of th[illegible]

Safran = Safran et al. 2016
SMRT contigs = CANU assembly of SMRT long-reads
HS1 = Hybrid Scaffold with BssSI
HS2 = Hybrid Scaffold with DLE-1
DE-HS = Dual Enzyme Hybrid Scaffold with BssSI and DLE-1
Final = Final assembly from DE-HS after removal of haplotigs

For all HS metrics including (column 1) and excluding (column 2) unscaffolded contigs

ran		HS1 (BssSI)		HS1 vs SMRT				HS2 (DLE-1)		HS2 vs HS1				DE-
(x)		Bionano Saphyr		Fold-change (x)				Bionano Saphyr		Fold-change (x)				Bionano
1.1			59.6			0.9			59.6			1.0		
	↑	8.327	10.789	1.6	↑	2.1	↑	17.321	25.954	2.1	↑	2.4	↑	23.843
	↑	0.106	1.15	1.1	↑	12.3	↑	0.094	2.336	0.89	↓	2.04	↑	0.11
	↑	1.34	1.18	1.02	↓	0.9	↓	1.34	1.15	1.001	↑	0.98	↓	1.35
.11			46.56			0.9			46.56			1.0		
	↑	104.6	91.8	1.02	↑	0.9	↓	104.7	89.7	1.001	↑	0.98	↓	105.6
	↓	3308	409	1.2	↓	9.5	↓	3317	211	0.997	↑	1.9	↓	3083
	↑	0.405	2.874	1.2	↑	8.5	↑	0.404	5.442	0.998	↓	1.9	↑	0.438
	↑	39.193	39.193	1.2	↑	1.2	↑	109.491	109.491	2.8	↑	2.8	↑	98.053
	↑	31	219	1.0	↑	8.5	↑	31	414	1.0	↑	1.9	↑	33.4

ıe *Gallus gallus* genome (GRCg6a) = 13146 bp

are reported

-HS	DE-HS vs HS2	Final	Final vs DE-HS	Final vs Safran
Saphyr	Fold-change (x)	No haplotigs	Fold-change (x)	Fold-change (x)
59.6	1.0	59.6	1.0	0.97
28.420	1.38 ↑ 1.10 ↑	25.954	1.09 ↑	668 ↑
2.12	1.22 ↓ 0.91 ↓	2.00	17.44 ↑	538 ↑
1.21	1.35 ↑ 1.05 ↑	1.21	0.90 ↑	1.1 ↑
46.56	1.0	46.56	1.0	0.99
94.4	1.009 ↑ 1.05 ↑	94.53	0.90 ↑	1.10 ↑
273	1.08 ↓ 0.77 ↑	364	8.47 ↓	275 ↓
4.428	1.09 ↑ 0.81 ↓	3.334	7.60 ↑	303 ↑
98.053	0.90 ↓ 0.90 ↓	98.053	1.00 ↑	134 ↑
336.8	1.09 ↑ 0.81 ↓	253.6	7.60 ↑	302 ↑

Figure 1

Click here to download Figure Figure 1.tif

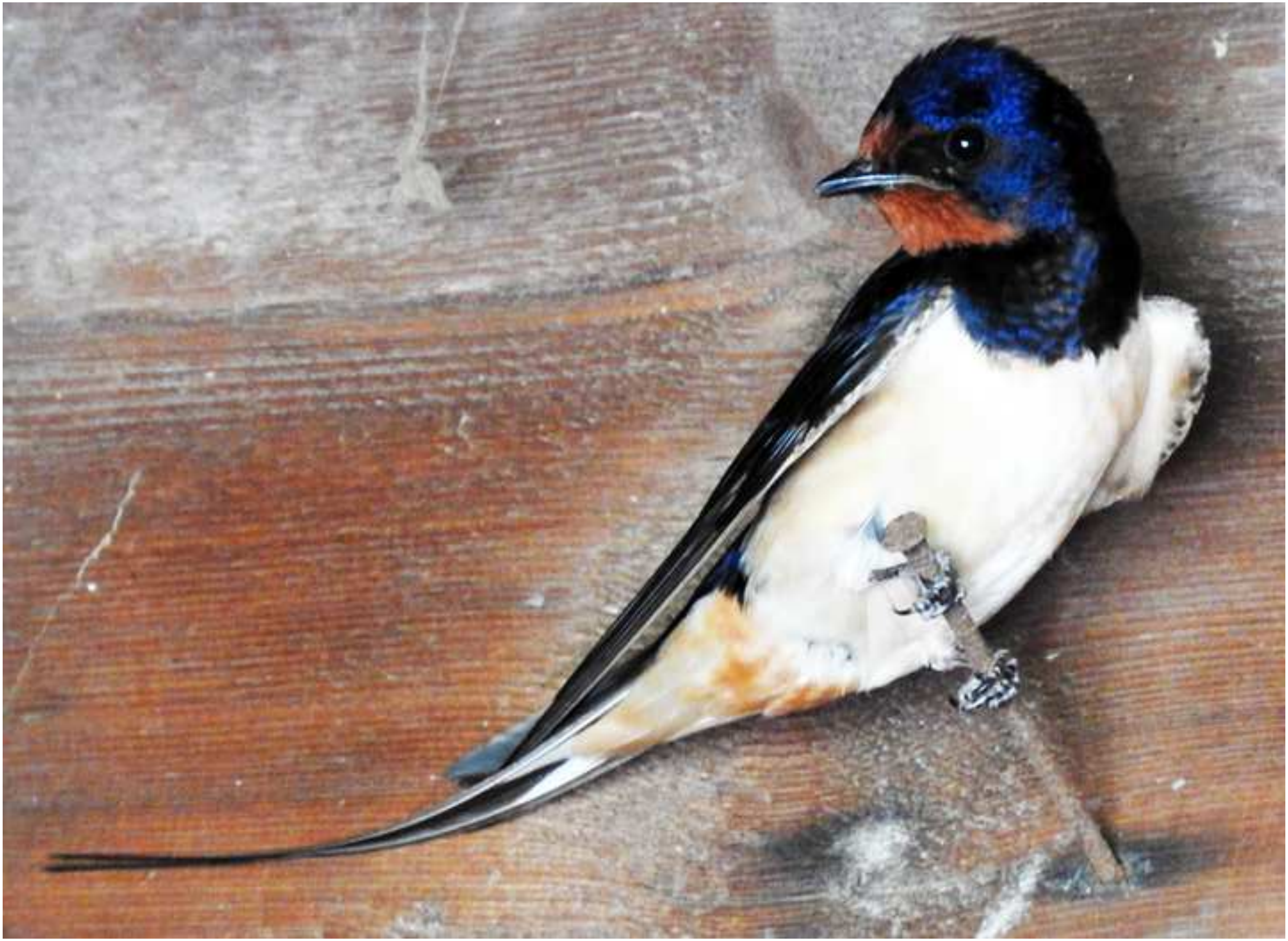

Figure 2

Click here to download Figure Figure 2.eps

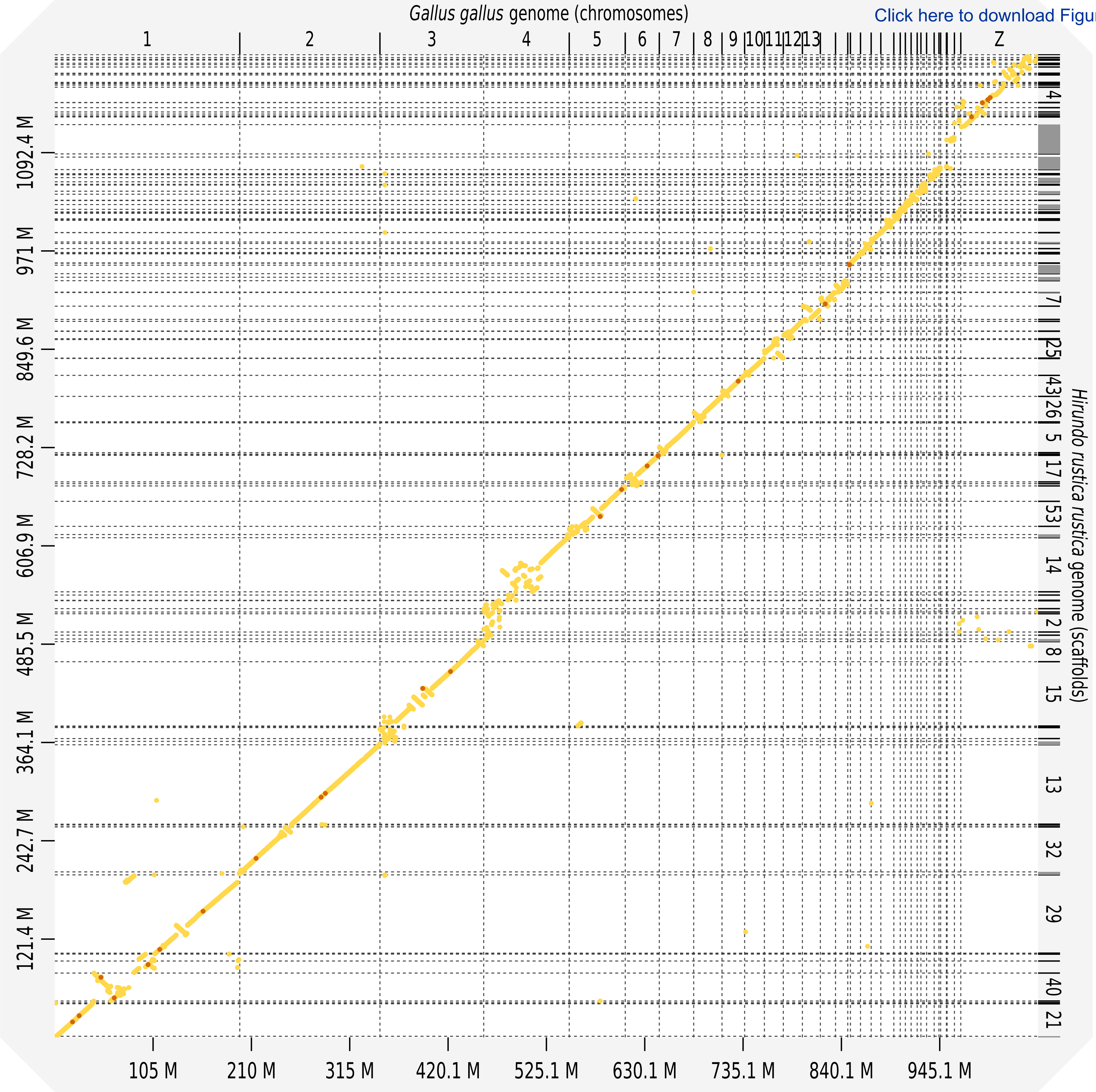

Supplementary Figure 1

Click here to access/download
Supplementary Material
Supplementary Figure 1.png

Supplementary Figure 2

Click here to access/download
Supplementary Material
Supplementary Figure 2.tif

Supplementary Figure 3

Click here to access/download
Supplementary Material
Supplementary Figure 3.png

Supplementary Figure 4

Click here to access/download
Supplementary Material
Supplementary Figure 4.png

Supplementary Figure 5

Click here to access/download
Supplementary Material
Supplementary Figure 5.png

Click here to access/download
Supplementary Material
GigaDBUploadForm_SWALLOW.xlsx
