# Supplementary material for: SMRT long reads and Direct Label and Stain optical maps allow the generation of a high-quality genome assembly for the European barn swallow (Hirundo rustica rustica)
Source: Gigascience. 2018 Nov 29;8(1):giy142. doi: 10.1093/gigascience/giy142 (PMC6324554; doi:10.1093/gigascience/giy142)
Supplement: giga-d-18-00272_revision_1.pdf [file giy142_giga-d-18-00272_revision_1.pdf]

GigaScience

SMRT long reads and Direct Label and Stain optical maps allow the generation of a high-quality genome assembly for the European barn swallow (Hirundo rustica rustica)

--Manuscript Draft--

Manuscript Number:	GIGA-D-18-00272R1
Full Title:	SMRT long reads and Direct Label and Stain optical maps allow the generation of a high-quality genome assembly for the European barn swallow (Hirundo rustica rustica)
Article Type:	Data Note
Funding Information:	California State Polytechnic University, Pomona — Dr. Andrea Bonisoli-Alquati
Abstract:	Background:<br>The barn swallow (Hirundo rustica) is a migratory bird that has been the focus of a large number of ecological, behavioural and genetic studies. To facilitate further population genetics and genomic studies, here we present a reference genome assembly for the European subspecies (H. r. rustica).<br><br>Findings:<br>As part of the Genome10K (G10K) effort on generating high quality vertebrate genomes, we have assembled a highly contiguous genome assembly using Single Molecule Real-Time (SMRT) DNA sequencing and several Bionano optical map technologies. We compared and integrated optical maps derived both from the Nick, Label, Repair and Stain and from the Direct Label and Stain (DLS) technologies. As proposed by Bionano, the DLS more than doubled the scaffold N50 with respect to the nickase. The dual enzyme hybrid scaffold led to a further marginal increase in scaffold N50 and an overall increase of confidence in the scaffolds. After removal of haplotigs, the final assembly is approximately 1.21 Gbp in size, with a scaffold N50 value of over 25.95 Mbp.<br><br>Conclusions:<br>This high-quality genome assembly represents a valuable resource for further studies of population genetics and genomics in the barn swallow, and for studies concerning the evolution of avian genomes. It also represents one of the very first genomes assembled by combining SMRT long-read sequencing with the new Bionano DLS technology for scaffolding. The quality of this assembly demonstrates the potential of this methodology to substantially increase the contiguity of genome assemblies.
Corresponding Author:	Giulio Formenti<br>University of Milan<br>Milano, Mi ITALY
Corresponding Author Secondary Information:	
Corresponding Author's Institution:	University of Milan
Corresponding Author's Secondary Institution:	
First Author:	Giulio Formenti, Graduate student
First Author Secondary Information:	
Order of Authors:	Giulio Formenti, Graduate student
	Matteo Chiara, Ph.D.
	Lucy Poveda, Ph.D.
	Kees-Jan Francoijs
	Andrea Bonisoli-Alquati, Assistant Professor
	Luca Canova

Powered by Editorial Manager® and ProduXion Manager® from Aries Systems Corporation

	Luca Gianfranceschi, Associate Professor
	David Stephen Horner, Associate Professor
	Nicola Saino, Full Professor
**Order of Authors Secondary Information:**	
**Response to Reviewers:**	Dear Editor,<br><br>First of all we would like to thank you and the reviewers for the prompt and detailed comments on our manuscript. We have addressed all the points raised, which resulted in improved clarity and appeal of the work.<br><br>Furthermore, during the initial review process, through communications with Erich Jarvis, the G10K Chair, regarding how to correctly acknowledge the G10K support of this work, we also understood that the Bionano DLS system has been utilized in several of their, as yet, unpublished genome assemblies. This fact, along with the correct acknowledgement to G10K approved by G10K Chair, has been inserted into the current version.<br><br>Below we provide detailed descriptions of the changes made as well as explanations of a couple of other unrequested modifications. The current version is slightly longer than the original submission, but we hope that you will agree that the extra length is justified by the content.<br><br>If you have any further queries regarding the submission, please do not hesitate to contact us.<br><br>---<br><br>Editor request:<br><br>Please ensure that there is sufficient methodological detail and we recommend that any relevant protocols are captured in protocols.io (there may be suitable protocols there for reuse or adapting, see our group collecting them https://www.protocols.io/groups/gigascience-journal). For validation we also recommend including a phylogenetic tree comparing where it fits some of the related sequenced species, especially in the light of the B10K project releasing so many bird genomes.<br><br>>We have now included an ML phylogenetic tree based on concatenated multiple gene alignments using our genome and those of some close relatives from the B10K project and other bird WGS projects. Our genome assembly places the barn swallow in the expected of the tree according to the literature (see text). Individual gene alignments have been added to the GigaDB submission associated with this work.<br><br>Reviewer reports:<br>Reviewer #1: ## General comments ##<br>The authors have produced a high-quality genome assembly of the European barn swallow, and important work that should allow the diverse research already done on the barn swallow to expand with a genomic basis.<br><br>The manuscript contains a lot of details about the methods, I would say much more than comparable manuscripts. This is very good, and very helpful for researchers interested in the methods the authors have used.<br><br>Be aware that the citations in the manuscript text link to a Paperpile reference, for instance this link: https://paperpile.com/c/R4nnD5/rZ3E. I cannot access the Google Document via that link. I guess this is the case for the bioRxiv version of this manuscript also. This will be removed in the processing of the manuscript before publication I guess, I just wanted you to be aware of it, because it can be a bit annoying.<br><br>>We removed any hyperlink from the document.

Specific comments

Abstract:
Line 9: This is a bit personal opinion, so you can ignore it, but I believe it is more accurate than what you have written: I prefer to refer to the digital version of a genome as a genome assembly. Genomes exist in individuals of a species, while genome assemblies exist in the computer. So you present a "high-quality genome assembly", and not a genome. You do this correct in line 32, but not necessary in the data description. I prefer "reference genome assembly", and not "reference genome", which would be something else.

>We modified the abstract according to the suggestion from the reviewer.

Data description:
Line 16: I find it strange that Molecular Ecology does not enforce release of this data (the Illumina based genome assembly). The editor of that paper should have held it back until the data was released. The scientific field does not progress as quickly as it could if people withhold data such as those guys do. It is great that you have released your data, as should be the default in all cases.

>We have once again formally requested the Journal to enforce the data release, explaining that are publishing a new HQ genome assembly for the barn swallow. We hope that this report will be taken into more serious consideration by the Journal.

Lines 77-80: You have a different font here for some reason.

>Solved.

Line 87: You have a comma instead of a punctuation mark in "5,2 Mbp".

>Solved.

Lines 173-176: Since you did this, I think you are correct in your conclusion, but the contigs might also just have not been placed. That is, the place the contigs (some of them at least) should have been was filled with a gap (Ns), and therefore the genomic location was in a way counted twice. First as the length of the contig, and then as the length of the gap. That could have explained some of the discrepancy, but since you found them to so similar to already assembled sequence, your explanation is better.

>We are unable to resolve with confidence haplotigs from potential gene duplication or indeed assembly errors for the time being. However, we draw the reviewer's attention to the last part of the results (lines 305-330), where we present specific arguments regarding repeats associated with individual chromosomes.

Line 179: The difference between the estimate and your genome assembly could be the centromeres and the telomeres, unless these are captured well in your assembly. I guess they might be the "poorly assembled repeats" you mention.

>We added centromeric and telomeric regions as examples (lines 245-246). We also include a more developed discussion of assembly size and conservation with other bird genomes which we hope will address several comments that were congruent between reviewers.

"Annotation of genes and repeats"
I think you describe most of the other methods well, but I would have liked a bit more detail in this section. How was the assembly repeat-masked? Did you use the built in bird repetitive elements, or create a de novo library?

>We used Chicken repeat models from RepBase. This is now clarified in the text (lines 263-270).

Did you just run Augustus with Gallus gallus as species? That is, you did not train Augustus on your genome? Or did you use Gallus gallus genes in the form of proteins or transcripts mapped to the assembly to train Augustus?

Powered by Editorial Manager® and ProduXion Manager® from Aries Systems Corporation

>Yes, we used the Gallus gallus gene models in Augustus rather than training on the swallow genome where we did not have sufficient cDNA data to reliably train Augustus. In the longer term, improved annotation incorporating experimental data will be provided. We believe that the text is reasonably clear on this.

You could mention how you found the PFAM domains. Was it with HMMER or InterProScan or something else? You did not use the annotation for anything else than comparing to Gallus gallus?

>We used PFAMScan (which includes HMMER). This is now clarified in the text.

Lines 211-214: I don't understand your reasoning here. If 52 of the 54 avian genome sequences contain these 40 genes, then would not that suggest that the 2 genome assemblies that do not contain them, are incomplete? Or are these 2 phylogenetically separate from the other 52 in some way?

>In the end we removed this comment, different possibilities could explain the observation (systematic difficulty of sequencing particular genomic regions with short read technology, non-universal presence of genes etc…). This discussion is interesting, but probably adds little to, and is outside the scope of the current work.

Line 226: Moore's law does not say anything about microchip complexity, but it is about the number of transistors on a integrated circuit.

>We have removed the slightly cliched reference to Moore's law.

Line 262: I don't think you explicitly compare your scaffolds to the chromosomes. I guess most of the large chromosomes would be found in 2-3 scaffolds. Is this correct? The synteny of birds is quite well conserved, so I think you could compare to chicken or zebra finch or something like that.

>We now provide a synthetic discussion of this issue as well as a table illustrating the numbers of scaffolds aligned to each chicken chromosome as well as the percentage of each chicken chromosome aligned (Table 2). We have also provided a slightly developed the discussion of sex chromosomes in the context of the overall genome size and the effect of long repeats such as centromeres and telomeres on our assembly.

Reviewer #2: This paper describes the sequencing and assembly of the European barn swallow using PacBio long reads and the latest technique for optical mapping. The result is a highly contiguous assembly that can benefit future genetic studies in barn swallows in particular and avian genomics in general.

The paper is well written and clear and I don't have any major concerns. Some general and specific comments follow below.

I was a bit surprised that the authors put so much focus on comparing their work with a previous Illumina-only based draft assembly (Safran et al 2016). It seems to me that the previous (not even publicly available) assembly was done very quick-and-dirty (using very few and short insert sizes, without any kind of curation at all) with the only purpose to facilitate SNP-calling with GBS data. Hence, it's not surprising at all that the new PacBio+Bionano assembly hundred-fold outperforms the previous draft, and I think the detailed comparison (both in text, table and supplementary table) is quite unnecessary, one single sentence mentioning this would have been enough.

>According to the reviewer's suggestion we have limited the emphasis to a single sentence mentioning the earlier genome assembly in the Context section of the manuscript (lines 82-83). In line with other specific comments from the reviewer with respect to this, we have also removed the comparisons with the earlier genome from tables.

The authors also mention that the old draft was based on a male and hence lack sequence from the W chromosome, but does not further comment on W scaffolds in

their new assembly. Especially since they point out the difference, I think it's appropriate to elaborate on this a bit more. The W chromosome is included in the latest Gallus gallus assembly so I assume it should be included in figure 2, but it's very hard to distinguish the different microchromosomes in this figure. An improvement could be adding a zoom-in on the W as a separate figure, and/or in the text mention the number of scaffolds that align to Gallus W including their summed up length. Is it less, the same, or even more than the 7Mb found in chicken?

>We have provided a brief discussion of sex chromosomes in the context of the overall genome size and the effect of long repeats in regions such as centromeres and telomeres on our assembly (lines 323-330).

Specific comments:
page 2, line 77-79 wrong font type for parts of the text

>Same as Reviewer#1. Solved.

page 4, line 105: "were expected", should be "was expected"

>Corrected.

page 7, line 178: "The final assembly is slightly smaller than the previously estimated genome size (1.28 Gbp) [9], possibly reflecting an imprecise older estimate and/or the possibility that some poorly assembled repeats were discarded in the final step described above." I'm sure both these suggestions could be true, but isn't it even more likely that some parts of the genome actually can't be assembled, not even with PacBio? I'm thinking mostly of centromeres, and the W chromosome which (just as the Y chromosome) has turned out to be very hard to assemble. For example in the chicken, karyotype stains show that chromosome W is about half the size of the 82 Mb long chromosome Z, but only 7Mb could be assembled. Also the (although few) fragmented or missing BUSCO genes suggest that not the entire genome is assembled. So to me it would have been more surprising if the barn swallow assembly equaled the "real" genome size.

>This comment from the reviewer mirrors that of the other reviewer. Both were correct and we believe that we addressed them when we state “The final assembly is slightly smaller than the previously estimated genome size (1.28 Gbp) [16], possibly reflecting an imprecise older estimate, and/or the possibility that some repeated sequences (e.g. centromeric and telomeric low complexity regions) were either collapsed in the initial assembly steps or discarded in the final haplotig purging step described above.” Please also see responses to previous related points.

page 10, line 262: "[..] as the fully assembled karyotype would have an N50 of ~ 90 Mbp". Is this based on chicken, or is the karyotype of barn swallow known? Please add a reference.

>This refers to the chicken assembly, as now made clear in the text (line 373).

Supplementary figure 2. Add explanations for the abbreviations "RFU" and "LM". To someone not familiar with this type of analysis, the vertical blue numbers and the orange vertical lines are a bit unclear, maybe an explanation can be added to the figure text? Why are there two different ">200000"? I assume the right part of the figure (darker and lighter bands) shows the same as the plot - is there some point in showing both, and if so, maybe they can be separated into a) and b)?

>Supplementary Figure 2 represents the typical output of the Femtopulse instrument. As suggested by the reviewer we added details and explanations to the caption of the figure and separated it into a) and b).

Supplementary figure 4. Parts of the boxplots lie outside the figure. Y axis label "Length" should also include "(bp)". In Supplementary figure 3 (bottom panel) it seems like one read is almost 160kb long, but here (in Supp fig 4) the scale ends at 150kb - is there any particular reason for removing that outlier?

Powered by Editorial Manager® and ProduXion Manager® from Aries Systems Corporation

	>We have corrected the figure according to the suggestions from the reviewer. The reason for the absence of 160 kbp long reads from the graph that the figure was produced with post-trimming reads as now indicated in the caption.<br><br>Supplementary figure 5. Please add labels to the axes also in the figure itself.<br><br>>We added the labels to the figure.<br><br>Supplementary table 1. This table contains so much information that it's a bit hard to grasp. Related to my first comment above, I don't think the comparison with the Safran assembly adds anything important here, and I recommend omitting the columns "Safran", "SMRT vs Safran" and "Final vs Safran" to make the table smaller. The new assembly is remarkable in itself and doesn't need this comparison to prove it.<br><br>>As already specified above, according to this and previous suggestion from the reviewer, we removed the comparison with the older assembly. We removed the comparison also from the main table in the text.<br><br>Also, I'm not sure I see the point in showing "Expected # of genes per contig"? It says that it's based on the average gene size based on chicken, but how is it actually calculated? Is the total gene count taken from chicken as well, or from the gene prediction? Does it at all take into account the uneven distribution of genes across the genome (mentioned in the main text, page 3 line 78-79)?<br><br>>The reviewer is completely correct in highlighting this issue. In fact the estimates were simply based on average gene length annotation in Chicken and did not take into account non-uniform gene density. This statistic was included to highlight the fact that most genes should be expected to be “completely” included within contigs (not necessarily the case for a short read assembly). However, given that in the current version of our manuscript we have greatly reduced the comparison to the previously reported Illumina WGS assembly, we therefore decided to omit these values from the current version.
**Additional Information:**	
**Question**	**Response**
Are you submitting this manuscript to a special series or article collection?	No
**Experimental design and statistics**<br><br>Full details of the experimental design and statistical methods used should be given in the Methods section, as detailed in our Minimum Standards Reporting Checklist. Information essential to interpreting the data presented should be made available in the figure legends.<br><br>Have you included all the information requested in your manuscript?	Yes
**Resources**<br><br>A description of all resources used, including antibodies, cell lines, animals	Yes

and software tools, with enough information to allow them to be uniquely identified, should be included in the Methods section. Authors are strongly encouraged to cite Research Resource Identifiers (RRIDs) for antibodies, model organisms and tools, where possible.<br><br>Have you included the information requested as detailed in our Minimum Standards Reporting Checklist?	
**Availability of data and materials**<br><br>All datasets and code on which the conclusions of the paper rely must be either included in your submission or deposited in publicly available repositories (where available and ethically appropriate), referencing such data using a unique identifier in the references and in the "Availability of Data and Materials" section of your manuscript.<br><br>Have you have met the above requirement as detailed in our Minimum Standards Reporting Checklist?	Yes

Powered by Editorial Manager® and ProduXion Manager® from Aries Systems Corporation

Manuscript

Click here to view linked References

Click here to access/download;Manuscript;Barn swallow genome Gigascience.docx

SMRT long reads and Direct Label and Stain optical maps allow the generation of a high-quality genome assembly for the European barn swallow (*Hirundo rustica rustica*)

Giulio Formenti* (giulio.formenti@unimi.it), Department of Environmental Science and Policy, University of Milan (Milan, Italy). ORCID: 0000-0002-7554-5991

Matteo Chiara* (matteo.chiara@unimi.it), Department of Biosciences, University of Milan (Milan, Italy). ORCID: 0000-0003-3983-4961

Lucy Poveda (lucy.poveda@fgcz.uzh.ch), Functional Genomics Center of Zurich, University of Zurich, (Zurich, Switzerland). ORCID: 0000-0002-5291-5582

Kees-Jan Francoijs (kfrancoijs@bionanogenomics.com), Bionano Genomics (San Diego, CA, USA). ORCID: 0000-0003-1360-5626

Andrea Bonisoli-Alquati (aalquati@cpp.edu), Department of Biological Sciences, California State Polytechnic University, Pomona (Pomona, CA, USA). ORCID: 0000-0002-9255-7556

Luca Canova (canova@unipv.it), Department of Biochemistry, University of Pavia (Pavia, Italy).

Luca Gianfranceschi (luca.gianfranceschi@unimi.it), Department of Biosciences, University of Milan (Milan, Italy). ORCID: 0000-0002-5011-7413

David Stephen Horner (david.horner@unimi.it), Department of Biosciences, University of Milan (Milan, Italy). ORCID: 0000-0002-6739-2657

Nicola Saino (nicola.saino@unimi.it), Department of Environmental Science and Policy, University of Milan (Milan, Italy). ORCID: 0000-0002-0230-3967

*These authors contributed equally to the work.

ABSTRACT

Background:

The barn swallow (*Hirundo rustica*) is a migratory bird that has been the focus of a large number of ecological, behavioural and genetic studies. To facilitate further population genetics and genomic studies, here we present a reference genome assembly for the European subspecies (*H. r. rustica*).

Findings:

As part of the Genome10K (G10K) effort on generating high quality vertebrate genomes, we have assembled a highly contiguous genome assembly using Single Molecule Real-Time (SMRT) DNA sequencing and several Bionano optical map technologies. We compared and integrated optical maps derived both from the Nick, Label, Repair and Stain and from the Direct Label and Stain (DLS) technologies. As proposed by Bionano, the DLS more than doubled the scaffold N50 with respect to the nickase. The dual enzyme hybrid scaffold led to a further marginal increase in scaffold N50 and an overall increase of confidence in the scaffolds. After removal of haplotigs, the final assembly is approximately 1.21 Gbp in size, with a scaffold N50 value of over 25.95 Mbp.

Conclusions:

This high-quality genome assembly represents a valuable resource for further studies of population genetics and genomics in the barn swallow, and for studies concerning the evolution of avian genomes. It also represents one of the very first genomes assembled by combining SMRT long-read sequencing with the new Bionano DLS technology for scaffolding. The quality of this assembly demonstrates the potential of this methodology to substantially increase the contiguity of genome assemblies.

Keywords: genome, barn swallow, third-generation sequencing, SMRT, long reads, Bionano, DLS, DLE-1, optical maps, single molecule.

Data Description

Context

The barn swallow is a passerine bird with at least eight recognized subspecies in Europe, Asia and North America. The European barn swallow (*Hirundo rustica rustica*, NCBI:txid333673) (Figure 1) breeds in a broad latitudinal range, between 63-68°N and 20-30°N [1]. Numerous evolutionary and ecological studies have focused on its biology, including its life history, sexual selection, and response to climate change. More recently, the barn swallow has become the focus of genetic studies on the divergence between subspecies and populations [2–4] and on the control of phenological traits [5–8]. Due to its synanthropic habits and its cultural value, the barn swallow is also a flagship species in conservation biology [1]. The availability of high-quality genomic resources, including a reference genome, is thus pivotal to further boost the study and conservation of this species.

Figure 1: the European barn swallow (*Hirundo rustica rustica*). Courtesy of Chiara Scandolara.

In 2016, Safran and coworkers reported the first draft of the genome for the American subspecies (*Hirundo rustica erythrogaster*) constructed from Illumina paired-end reads [2]. However, it has not been possible to analyze this assembly as neither the raw nor the assembled data were publicly available at the time of preparation of the current manuscript [2].

Here we have employed two single-molecule technologies, Single Molecule Real Time (SMRT) Third-Generation Sequencing (TGS) from Pacific Biosciences (Menlo Park, California, USA) and optical mapping from Bionano Genomics (San Diego, California, USA), to produce a state-of-the-art high-quality genome assembly for the European subspecies. For optical mapping we labelled DNA molecules both with one of the original Nick, Label, Repair and Stain (NLRS) nickases (enzyme Nb.BssSI) and with the new Direct Label and Stain (DLS) approach (enzyme DLE-1). The latter technique was officially released in February 2018 and avoids nicking and subsequent cleavage of DNA molecules during staining [9]. We show that DLS allows a considerable improvement of scaffold contiguity with respect to the nickase tested, consistent with Bionano's claim. Furthermore,

the "dual enzyme" approach affords additional support for scaffold junctions. This genome assembly is among the first to incorporate DLS and SMRT sequencing data, providing assembly contiguity metrics well in excess of those specified for "Platinum genomes" by the Vertebrate Genomes Project (VGP) [10,11]. While this article was under review, the Vertebrate Genomes Project released 15 genome assemblies that incorporate SMRT and DLS data among others, including the hummingbird and Kakapo, with comparable results (Bioproject PRJNA489243) [12].

Blood sample collection

The blood used as a source of DNA was derived from a minimally invasive sampling performed on a female individual of approximately two years of age during May 2017 in a farm near Milan, in Northern-Italy (45.4N 9.3E). Blood was collected in heparinized capillary tubes. Three hours after collection, the sample was centrifuged to separate blood cells from plasma, and then stored at -80°C.

DNA extraction and quality control for SMRT library preparation

DNA extraction was performed on blood cells portion of centrifuged whole blood containing nucleated erythrocytes and leukocytes with the Wizard genomic DNA purification kit (Promega, Cat. No. A1125), using the protocol for tissue (not human blood). This kit employs a protocol similar to the classical Phenol/Chloroform DNA extraction, with no vortexing steps after cell lysis. After purification, DNA quality and concentration were assessed by Nanodrop (Thermo Fisher Scientific, Cat. No. ND-1000), and subsequently by Pulsed Field Gel Electrophoresis (PFGE). Detectable DNA was over 23 kbp in size, with the vast majority over 50 kbp and even over 200 kbp (Supplementary Figure 1). PFGE quality results were further confirmed by capillary electrophoresis on FEMTO Pulse instrument (AATI, Cat. No. FP-1002-0275) (Supplementary Figure 2). DNA was stored at -80°C and shipped to the sequencing facility on dry ice.

SMRT library preparation and sequencing

SMRTbell Express Template Prep Kit (Pacific Biosciences, Cat. No. 101-357-000) was used to produce the insert library. Input genomic DNA (gDNA) concentration was measured on a Qubit Fluorometer dsDNA Broad Range (Life Technologies, Cat. No. 32850). 10 µg of gDNA was mechanically sheared to an average size distribution of 40-50 kbp, using a Megaruptor Device (Diagenode, Cat. No. B06010001). FEMTO Pulse capillary electrophoresis was employed to assess the size of the fragments. 5 µg of sheared gDNA was DNA-damage repaired and end-repaired using polishing enzymes. Blunt-end ligation was used to create the SMRTbell template. A Blue Pippin device (Sage Science, Cat. No. BLU0001) was used to size-select the SMRTbell template and enrich for fragments > 30 kbp, excluding the first two cells for which the library was enriched for fragments > 15 kbp. The size-selected library was checked using FEMTO Pulse and quantified on a Qubit Fluorometer. A ready to sequence SMRT bell-Polymerase Complex was created using the Sequel binding kit 2.0 (Pacific Biosciences, Cat. No. 100-862-200). The Pacific Biosciences Sequel instrument was programmed to sequence the library on 18 Sequel SMRT Cells 1M v2 (Pacific Biosciences, Cat. No. 101-008-000), taking one movie of 10 hours per cell, using the Sequel Sequencing Kit 2.1 (Pacific Biosciences, Cat. No. 101-310-400). After the run, sequencing data quality was checked via the PacBio SMRT Link v5.0.1 software using the “run QC module”. An average of 3.7 Gbp (standard deviation: 1.7) were produced per SMRT cell (average N50 = 25,622 bp), with considerable improvements between the average 15 kbp library and the 30 kbp library (see Supplementary Figure 3 for more detailed statistics). We observed a wide distribution in the GC content of reads (Supplementary Figure 4). This is likely explained by the presence in avian genomes of three classes of chromosomes: macrochromosomes (50-200 Mbp, 5 in chicken), intermediate chromosomes (20-40 Mbp, 5 in chicken) and microchromosomes (12 Mbp on average, 28 in chicken). These last account for only 18% of the total genome but harbour ~31% of all chicken genes, have higher recombination rates and higher GC contents on average [13].

Assembly of SMRT reads

The final assembly of long reads was conducted with software CANU v1.7 (Canu, RRID:SCR_015880)[14] using default parameters except for the "correctedErrorRate" which was set at 0.075. The assembly processes occupied 3,840 CPU hours and 2.2 Tb of RAM for read correction, 768 CPU hours and 1.1 Tb of RAM for the trimming steps, and 3280 CPU hours and 2.2 Tb of RAM for the assembly phase. The long-read assembly contained 3,872 contigs with a N50 of 5.2 Mbp for a total length of 1311.7 Mbp (Table 1 and Supplementary Table 1). Final polishing was performed using the Arrow v2.10 software (Pacific Biosciences) and resulted in final coverage of 45.4x.

Cell count and DNA extraction for optical mapping

High-molecular weight (HMW) DNA was extracted from 7-8 µl of the cell portion from the same blood sample used for SMRT sequencing with the Blood and Cell Culture DNA Isolation kit (Bionano Genomics, Cat. No. RE-016-10). HMW DNA was extracted by embedding cells in low melting temperature agarose plugs that were incubated with Proteinase K (Qiagen, Cat. No. 158920) and RNAseA (Qiagen, Cat. No. 158924). The plugs were washed and solubilized using Agarase Enzyme (Thermo Fisher Scientific, Cat. No. EO0461) to release HMW DNA and further purified by drop dialysis. DNA was homogenised overnight prior to quantification using a Qubit Fluorometer.

***In silico* digestion**

The genome assembly obtained with CANU was *in silico* digested using Bionano Access software to test whether the nicking enzyme (Nb.BssSI), with recognition sequence (CACGAG), and the non-nicking enzyme DLE-1, with recognition sequence (CTTAAG), were suitable for optical mapping in our bird genome. An average of 16.9 nicks/100 kbp with a nick-to-nick distance N50 of 11,708 bp were expected for Nb.BssSI, while DLE-1 was found to induce 19.1 nicks/100 kbp with a nick-to-nick distance N50 of 8,775 bp, both in line with manufacturer's requirements.

DNA labeling for optical mapping

For NLRS, DNA was labelled using the Prep DNA Labeling Kit-NLRS according to manufacturer's instructions (Bionano Genomics, Cat. No. 80001). 300 ng of purified gDNA was nicked with

Nb.BssSI (New England Biolabs, Cat. No. R0681S) in NEB Buffer 3. The nicked DNA was labelled with a fluorescent-dUTP nucleotide analog using Taq DNA polymerase (New England BioLabs, Cat. No. M0267S). After labeling, nicks were ligated with Taq DNA ligase (New England BioLabs, Cat. No. M0208S) in the presence of dNTPs. The backbone of fluorescently labelled DNA was counterstained overnight with YOYO-1 (Bionano Genomics, Cat. No. 80001).

For DLS, DNA was labelled using the Bionano Prep DNA Labeling Kit-DLS (Cat. No. 80005) according to manufacturer's instructions. 750 ng of purified gDNA was labelled with DLE labeling Mix and subsequently incubated with Proteinase K (Qiagen, Cat. No. 158920) followed by drop dialysis. After the clean-up step, the DNA was pre-stained, homogenised and quantified using on a Qubit Fluorometer to establish the appropriate amount of backbone stain. The reaction was incubated at room temperature for at least 2 hours.

Generation of optical maps

NLRS and DLS labelled DNA were loaded into a nanochannel array of a Saphyr Chip (Bionano Genomics, Cat. No. FC-030-01) and run by electrophoresis each into a compartment. Linearized DNA molecules were imaged using the Saphyr system and associated software (Bionano Genomics, Cat. No. 90001 and CR-002-01).

In the experiment with Nb.BssSI, molecule N50 was 0.1298 Mbp for molecules above 20 kbp and 0.2336 Mbp for molecules above 150 kbp - with an average label density of 11.8/100 kbp for molecules above 150 kbp. Map rate was 38.9% for molecules above 150 kbp. Effective coverage was 28.2x. In the experiment with DLE-1, molecule N50 was 0.2475 Mbp for molecules above 20 kbp and 0.3641 Mbp for molecules above 150 kbp - with an average label density of 15.7/100 kbp for molecules above 150 kbp. Map rate was 56.4% for molecules above 150 kbp. Effective coverage was 30.6x. Using both Nb.BssSI and DLE-1, label metrics were in line with the manufacturer's expectations.

Assembly of optical maps

The *de novo* assembly of the optical maps was performed using the Bionano Access v1.2.1 and Bionano Solve v3.2.1 software. The assembly type performed was the “non-haplotype” with “no extend split” and “no cut segdups”. Default parameters were adjusted to accommodate the genomic properties of the barn swallow genome. Specifically, given the size of the genome, the minimal length for the molecules to be used in the assembly was reduced to 100 kbp, the “Initial P-value” cut off threshold was adjusted to 1×10^{-10} and the P-value cut off threshold for extension and refinement was set to 1×10^{-11} according to manufacturer's guidelines (default values are 150 kbp, 1×10^{-11} and 1×10^{-12} respectively).

A total of 233,450 (of 530,527) NLRS-labelled molecules (N50 = 0.2012 Mbp) were aligned to produce 2,384 map fragments with an N50 of 0.66 Mbp for a total length of 1338.6 Mbp (coverage = 32x). 108,307 (of 229,267) DLE-1 labelled input DNA molecules with a N50 of 0.3228 Mbp (theoretical coverage of the reference 48x) produced 555 maps with a N50 length of 12.1 Mbp for a total length 1299.3 Mbp (coverage = 23x).

Hybrid scaffolding

Single and dual enzyme Hybrid Scaffolding (HS) was performed using Bionano Access v1.2.1 and Bionano Solve v3.2.1. For the dual enzyme and DLE-1 scaffolding, default settings were used to perform the HS. For Nb.BssSI the “aggressive” settings were used without modification. The NLRS HS had an N50 of 8.3 Mbp (scaffold only N50 = 10.8 Mbp) for a total length of 1,338.6 Mbp (total length of scaffolded contigs = 1,175.3 Mbp) and consisted of 409 scaffolds and 2,899 un-scaffolded contigs. The DLS HS had scaffold N50 of 17.3 Mbp (scaffold only N50 = 25.9 Mbp) for a total length of 1,340.2 Mbp (total length of scaffolded contigs = 1,148.4 Mbp) and consisted of 211 scaffolds and 3,106 un-scaffolded contigs. Dual enzyme HS (incorporating both NLRS and DLS maps) resulted in an assembly with N50 of 23.8 Mbp (scaffold only N50 = 28.4 Mbp) for a total length of 1,351.8 Mbp (total length of scaffolded contigs = 1,208.8 Mbp) and consisted of 273 scaffolds and 2,810 un-scaffolded contigs. During the automatic conflict resolution in the dual enzyme HS, 185 SMRT contigs were cut, as Bionano maps confidently indicated mis-assemblies of the SMRT reads. Conversely, 117 Bionano maps were cut, indicating that the chimeric score did not provide sufficient

confidence to cut the assembly based on SMRT contigs. Of 3,872 SMRT contigs, 1,243 (32%) were anchored in the Bionano maps (of which 990 were anchored in both NLRS and DLS maps). 226 and 56 were anchored in NLRS and DLS maps respectively. 2,810 maps could not be anchored at all.

Purge of haplotigs and final assembly

Notably, all hybrid assemblies were somewhat larger than the expected genome size, and in all cases, the N50 of un-scaffolded contigs was extremely low (0.06 Mbp for the dual enzyme hybrid assembly). We hypothesized that a significant proportion of these small contigs might represent divergent homologous haplotigs that were assembled independently [15]. Similarity searches were consistent with this possibility as almost 95% of the contigs that were not scaffolded in the dual enzyme hybrid assembly showed > 98% identity to scaffolded contigs over 75% of their length or more. These contigs were discarded, resulting in a final assembly (Table 1 and Supplementary Table 1 for detailed statistics) of 1.21 Gbp (N50 = 25.9 Mbp) made up of 273 dual enzyme hybrid scaffolds (N50 = 28.42 Mbp) and 91 un-scaffolded contigs (N50 = 0.0644 Mbp). The final assembly is slightly smaller than the previously estimated genome size (1.28 Gbp) [16]. This potentially reflects an imprecise older estimate, and/or the possibility that some repeated sequences (e.g. centromeric and telomeric low complexity regions) were either collapsed in the initial assembly steps or discarded in the final haplotig purging step described above. The average SMRT read coverage for the genome assembly was 34.15X (implying a theoretical QV of over 40). Supplementary Figure 5 provides a summary of observed sequence coverage depth.

Assembly metrics for contigs and final scaffolds in our European barn swallow genome.

	**SMRT contigs[1]**	**Final assembly[2]**
Species	*H. r. rustica*	
Starting raw data (Gbp)	66.4	59.6
N50 (bp)	5189284	25954216
N90 (bp)	85340	2002624

Total size (Gbp)	1.31	1.21
Theoretical genome coverage*	52x	47x
% genome coverage*	102.6	94.5
# of contigs/scaffolds	3872	364
Avg contig/scaffold length (bp)	338782	3334461
Longest contig/scaffold (bp)	33230000	98053015

Table 1: [1] SMRT reads assembled using CANU v1.7 [14]. [2] SMRT contigs assembled with CANU and scaffolded using Bionano dual enzyme HS, with haplotigs removed as detailed in the text. *Based on a barn swallow genome size estimate of 1.28 Gbp [16].

Annotation of genes and repeats

With respect to mammals, avian genomes generally contain relatively low proportions of repetitive sequences and show strong mutual synteny [17]. This appears to be the case for the barn swallow genome. In particular, 7.11% of the final assembly was annotated as repetitive using WindowMasker [18] and RepeatMasker (RepeatMasker, RRID:SCR_012954)[19]. The major contributors to annotated repeats were L2/CR1/Rex LINE elements (3.37%), retroviral LTRs (1.59%) and simple repeats (1.56%).

Repeats were soft-masked prior to *de novo* gene prediction using Augustus (Augustus, RRID:SCR_008417)[20] with *Gallus gallus* gene models. In all, 35,644 protein coding genes were predicted, of which 9,189 were overlapped by more than 30% of their size with repetitive genomic elements. Of the remaining 26,455 predicted protein coding genes, 24,331 harboured a PFAM protein domain (as identified by PfamScan v1.6 [21]). Simple similarity searches based on blastp [22] (with default parameters) suggested that 17,895 of the predicted protein coding genes have a best reciprocal blast hit with gene models derived from *G. gallus* GRCg6a assembly (as available from [23]), while 2,927 of the proteins predicted by Augustus did not show any significant match (e-value <= 1×10^{-15}, identity > 35%).

BUSCO genes and phylogenetic reconstruction

Of a total of 4,915 conserved bird Benchmarking with Universal Single-Copy Orthologs (BUSCO) groups (BUSCO , RRID:SCR_015008)[24] sought, 4,598 (93.6%) were complete (and mostly single-copy, 4,521 overall - 92.0%, while 77 were duplicated - 1,6%), 192 (3.9%) were fragmented and 125 (2.5%) were missing. The percentage of contiguously assembled BUSCO genes is consistent with recent results with Anna's hummingbird (*Calypte anna*) and the Zebra Finch (*Taeniopygia guttata*) [15].

Protein sequences inferred from coding sequences identified by BUSCO v3 as barn swallow orthologs of universal avian single copy genes were aligned to passerine orthologs present in orthoDB v9.0 [25] when all represented passerines had an annotated ortholog. A total of 3,927 protein alignments were generated using the software muscle v3.8.31 [26] with default settings. Software GBlocks v0.91b [27] with default settings apart from allowing gaps in final blocks was used to exclude low quality alignment regions. Trimmed protein alignments were concatenated to produce a supergene alignment with 1,707,664 amino acid positions. Maximum-Likelihood (ML) phylogenetic inference and estimation of aLRT branch support indexes were performed using the software PhyML v3.0 [28], with the LG substitution matrix [29] incorporating 4 variable and 1 invariable gamma distributed substitution rate categories. Distance bootstrap proportions (100 replicates) were estimated using the BioNJ method with the Kimura protein distance correction as implemented in the software SeaView v4.6.5 [26]. ML phylogenetic analysis of concatenated protein sequence alignments yielded a robustly supported topology (Supplementary Figure 6) that is consistent with previous phylogenomic studies [30,31] as well as with gene-level phylogenies [32,33].

Synteny with the Chicken genome

Alignment of the final assembly with the most recent assembly of the chicken genome (GRCg6a) using D-Genies [34] indicates high levels of collinearity between these two genomes with a limited number of intra-chromosomal rearrangements (Figure 2). The high level of collinearity between independently assembled and scaffolded sequences provides circumstantial support for the quality of

both the contigs and the hybrid scaffolds, and is consistent with previous observations of high levels of synteny and minimal inter-chromosomal rearrangements among birds [17].

Figure 2: Alignment of the final assembly with the published chromosome-level assembly of the chicken (*G. gallus*) genome GRCg6a using D-Genies [34]. Light to dark yellow dots indicate progressively higher similarity between sequences.

Overall, 90.44% of the chicken assembly can be uniquely aligned to regions in the barn swallow assembly. Table 2 shows for each chicken chromosome (assembly GRCg6a) the number of barn swallow scaffolds aligning uniquely (by best reciprocal BLAST analysis) as well as the percentage of the chicken chromosome involved in alignments. Together with the synteny plot shown in Figure 2, these data indicate that a high proportion (>85%) of most barn-swallow autosomes are likely assembled in less than 10 scaffolds. Indeed several chromosomes are likely assembled as single scaffolds. However, some alignments of chicken chromosomes to the barn swallow assembly are either notably more fragmented or partial. In particular, a large proportion of chicken chromosomes 1 and 4 are represented in unique alignments with the barn swallow assembly. However, for both these chromosomes a number of rearrangements are implied (Figure 2), in line with previous comparisons between the chicken genome and those of other Passeriformes [35–37]. Unambiguous matches between chicken chromosome 16 (2.84 Mb in the chicken assembly GRCg6a, 16 Mb according to flow karyotyping [38]) and the barn swallow assembly were scarce, consistent both with previous reports of difficulties in assembling this chromosome [35,39], likely due to the unusual gene distribution, presence of rRNA repeats, and the polymorphic and often polygenic MHC loci [40] on this chromosome. Similarly, chromosome 31, for which RepeatMasker identified 3.57 Mb (58% of the GRCg6a chromosome assembly) as repeats, was also assembled in a rather fragmented manner in the barn swallow.

Of the sex chromosomes, chicken chromosome Z sequences are well represented, if somewhat fragmented in the barn swallow assembly. The discontinuous assembly of this chromosome is likely related to the widespread presence of repeats [41,42]. For the chicken W chromosome (6.81 Mb in the

GRCg6a chromosome assembly, 43 Mb according to flow karyotyping [38]), apparent orthologs of 45 (of 53 single copy genes annotated on the W chromosome of in the *G. gallus* assembly) were identified in the barn swallow genome, although only 46% of the assembled chicken chromosome found best reciprocal BLAST matches. Indeed, Avian W chromosomes are gene-poor and contain long, lineage specific repeats [43,44], complicating both assembly and comparative analyses.

Alignment between the *G. gallus* GRCg6a and barn swallow genome assemblies.

Chromosome	N. of uniquely aligned scaffolds	Size in GRCg6a assembly (Mbp)	Covered scaffolds from our assembly
1	9	197.61	92.83
2	6	149.68	88.10
3	7	110.84	91.70
4	15	91.32	94.48
5	3	59.81	89.91
6	2	36.37	91.87
7	1	36.74	90.18
8	1	30.22	90.26
9	1	24.15	92.65
10	2	21.12	87.05
11	2	20.2	89.74
12	3	20.39	92.40
13	2	19.17	90.54
14	1	16.22	91.01
15	1	13.06	91.62
16	3	2.84	46.10
17	1	10.76	93.57
18	3	11.37	96.86
19	1	10.32	88.22
20	2	13.9	92.45
21	1	6.84	95.03
22	3	5.46	85.47
23	2	6.15	87.65
24	1	6.49	92.78
25	1	3.98	90.50
26	1	6.06	94.06
27	3	8.08	96.75
28	3	5.12	94.61
30	13	1.82	72.81
31	4	6.15	28.14
32	6	0.73	95.35
33	5	7.82	92.63
W	5	6.81	45.90
Z	34	82.53	89.82

Table 2: For each chicken chromosome the number of scaffolds aligning uniquely as well as the percentage of the chicken chromosome involved in alignments are reported.

Conclusion

Short-read NGS (now known as Second Generation Sequencing, or SGS) technologies have allowed the production of cost-effective genome drafts for many birds and other vertebrate species [30,45,46]. However, the reduction in genome sequencing costs has typically come at the price of compromises in contiguity and accuracy of assemblies with respect to earlier efforts based on Sanger reads and extensive physical mapping [47]. Many limitations of SGS-based assemblies stem from the occurrence of long sequence repeats. In many animal species, transposons are frequently located in introns [48] and the presence of large gene families of closely related paralogs can lead to the existence of long "genic" repeats. Accordingly, even apparently contiguous genic regions can feature juxtaposition of paralogous gene fragments [15]. Given the inception of large scale sequencing initiatives aiming to produce genome assemblies for a wide range of organisms [49–52], it is critical to identify combinations of sequencing and scaffolding approaches that allow the cost effective generation of genuinely high-quality genome assemblies [10]. While exhibiting higher rates of single-base errors than some SGS approaches, TGS technologies, including SMRT sequencing, offer read-lengths unparalleled by SGS or Sanger sequencing [53]. Moreover, recent and ongoing improvements in TGS methods are rapidly reducing the "per-base" cost of TGS data compared to that of SGS. On the other hand, as an alternative to scaffolding with long insert mate-pairs [54] or to chromatin proximity ligation sequencing [55], contiguity and accuracy of long-read-based assemblies can be further improved by optical mapping. This relies on nanoscale channels that can accommodate thousands of single, ultralong (>200 kbp) double-stranded DNA filaments in parallel, subsequently stained to recognize specific 6-7 bp long motifs [56]. The combination of long reads and optical maps has already proven invaluable to produce high-quality genome assemblies, even in the case of particularly complex genomes [57]. Here, using only SMRT sequencing and Bionano optical maps we have produced a high-quality and contiguous genome for the barn swallow. With respect to a

previously reported SGS-based assembly of the American barn swallow genome using a comparable amount of raw data [2], even the contigs generated from long-read sequencing alone show a 134-fold increase in N50, similar to the increase recently obtained for the Anna's hummingbird genome using the same technologies [15]. Furthermore, the 1.6 fold change in scaffold N50 attained by Bionano NLRS hybrid scaffolding before removal of haplotigs is comparable with results obtained by other genome assemblies that have employed this method [58]. Strikingly, the new DLS method greatly outperformed the NLRS system, providing a 3.3 fold increase of N50 (before removal of haplotigs). Moreover, incorporation of both labelling systems into the hybrid scaffolding yielded a final assembly showing 5-fold improvement of the N50 with respect to the original SMRT assembly, simultaneously providing "independent" validation of many scaffold junctions. We note that the presence of numerous microchromosomes in avian genomes restricts the final N50 value potentially attainable for the assembly, as for example the fully assembled karyotype of the chicken genome assembly (GRCg6a) would have an N50 of ~ 90 Mbp. Yet, after removal of putative haplotigs, our genome assembly contiguity metrics meet the high standards of the VGP consortium "Platinum Genome" criteria (contig N50 in excess of 1 Mbp and scaffold N50 above 10 Mbp) [10]. Accordingly, we believe that the data presented here, while attesting to the effectiveness of SMRT sequencing combined with DLS optical mapping for the assembly of vertebrate genomes, will provide an invaluable asset for population genetics and genomics in the barn swallow and for comparative genomics in birds.

Re-use Potential

Future directions for the barn swallow genome will include further scaffolding using a G10K VGP approach, the phasing of the assembly to generate extended haplotypes, a more thorough gene annotation using RNA/IsoSeq sequencing data, detailed comparisons with the genome of the North American subspecies, *H. r. erythrogaster*, studies on the genomic architecture of traits under natural and sexual selection, and the re-evaluation of data from population genetics studies conducted in this species (as it was shown that the availability of a high quality genome may change the interpretation of some results), as well as characterization of the epigenetic landscape.

Availability of supporting data

Sequencing data supporting the results of this article are in the GenBank repository under Bioproject PRJNA481100, and the optical maps, annotations, and other data are available in the *GigaScience* GigaDB repository[59].

Competing interests

Kees-Jan Francoijs is currently employed at Bionano Genomics (San Diego, CA, USA). All other authors declare no competing interest.

Funding

Funding to A.B.-A. was provided by Cal Poly Pomona College of Science.

Authors' contributions

G.F, N.S., A.B.-A., L.G., D.S.H, M.C. and L.C. conceived the project and designed the experiments; G.F. performed DNA extraction and quality control; M.C. carried out CANU assembly, gene and repeat annotation. D.S.H., M.C. and L.G. performed other bioinformatics analyses; L.P. conducted the optical mapping; K.J.F. produced the hybrid scaffolds; G.F., D.S.H, M.C., N.S. and L.C. drafted the manuscript. All authors edited and contributed to the manuscript.

Acknowledgements

We thank Manuela Caprioli for support in field work, sample collection, DNA extraction and quality control as well as Dr. Elena Galati for support in PFGE quality control. We also thank The Functional Genomics Center of Zurich, where SMRT sequencing and optical mapping were carried out, and particularly Andrea Patrignani for SMRT sequencing. We are thankful to the Genome 10K Council and to all members of the Consortium for the support in obtaining early access to the DLS technology. We are particularly thankful to the G10K Chair Prof. Erich Jarvis, also for his helpful comments to the manuscript. We thank Chiara Scandolara for the barn swallow picture used for Figure 1. We

acknowledge the support of ELIXIR-IT and CINECA (HPC@CINECA) for provision of computational resources for SMRT read assembly. We thank the reviewers who helped us to considerably improve the first version of this manuscript.

Ethics approval

The blood sample used to generate the genomic data derived from a minimally invasive sampling on a single individual. Appropriate consent was obtained from the local authorities (Regione Lombardia).

Additional files

Supplementary Figure 1 (Supplementary Figure 1.png)

PFGE on a 1x agarose gel run for 18 hours at 160 mV. The two lowest overlapping bands in lane 1 represent yeast chromosomes of 230 kbp and 270 kbp, respectively. Lane 2 contains 1kb DNA ladder (highest 10 kbp), lane 3 and 4 the undigested lambda phage (50 kbp) and lane 5 digested lambda (upper band 23 kbp). Lane 7 contains the sample used in the study.

Supplementary Figure 2 (Supplementary Figure 2.tif)

FEMTO Pulse capillary electrophoresis results from software PROSize Data Analysis (AATI) for the DNA sample used in the study. a) Quantity by fragment size plot. The software algorithm identifies the peaks of major fluorescence change (defined within the range of 2 orange bars) and assign a size value to them (blue numbers). The purple dashed line represents the 50 kpb cutoff. RFU = Relative Fluorescence Unit. LM = Lower Marker. b) Virtual gel. Note that DNA > 200 kbp is above the detection range of the instrument and is conventionally labelled as > 200 kbp.

Supplementary Figure 3 (Supplementary Figure 3.png)

Summary statistics for each SMRT cell employed.

Supplementary Figure 4 (Supplementary Figure 4.png)

GC content distribution in all sequence reads after CANU trimming.

Supplementary Figure 5 (Supplementary Figure 5.tif)

Cumulative coverage distribution of the final (de-haplotyped) assembly of the barn swallow genome. Coverage is indicated on the X axis. Red lines are used to display the proportion of the genome covered by more than 10, 20, 30, 40, 50 or 60 reads respectively.

Supplementary Figure 6 (Supplementary Figure 6.eps)

Maximum likelihood phylogenetic tree based on a multiple alignment of 3,927 gene orthologs in passerine species. The scale bar indicates inferred changes per site, aLRT support values and neighbor joining bootstrap values (100 replicates) are shown on branches.

Supplementary Table 1 (Supplementary Table 1.xlsx)

Comparison of assembly metrics for contigs and scaffolds between different assemblies. In hybrid scaffolds, the first column refers to assemblies including the un-scaffolded contigs while the second column only includes scaffolded contigs metrics. The estimated genome size of 1.28 Gbp is from [16]. Average gene size was estimated according to the latest available annotation of the *G. gallus* genome (GRCg6a).

List of abbreviations

BUSCO, Benchmarking with Universal Single-Copy Orthologs; CPU, central processing unit; DLS, Direct Label and Stain; G10K, Genome 10K (10,000 vertebrate genome project); Gbp, Giga base-pairs; HMW, High Molecular Weight; HS, Hybrid Scaffold; Mbp, Mega base-pairs; MHC, major histocompatibility complex; NGS, Next Generation Sequencing; NLRS, Nick, Label, Repair and Stain; N50, the shortest sequence length at 50% of the genome; N90, the shortest sequence length at 90% of the genome; PFGE, Pulsed Field Gel Electrophoresis; QV, Quality Value; SGS, Second Generation Sequencing; SMRT, Single Molecule Real-Time; TGS, Third Generation Sequencing; VGP, Vertebrate Genomes Project.

References

1. Turner A. The barn swallow. T & AD Poyser, London; 2006.

2. Safran RJ, Scordato ESC, Wilkins MR, Hubbard JK, Jenkins BR, Albrecht T, et al. Genome-wide differentiation in closely related populations: the roles of selection and geographic isolation. Mol Ecol. 2016;25:3865–83. http://dx.doi.org/10.1111/mec.13740

3. von Rönn JAC, Shafer ABA, Wolf JBW. Disruptive selection without genome-wide evolution across a migratory divide. Mol Ecol. 2016;25:2529–41. http://dx.doi.org/10.1111/mec.13521

4. Scordato ESC, Wilkins MR, Semenov G, Rubtsov AS, Kane NC, Safran RJ. Genomic variation across two barn swallow hybrid zones reveals traits associated with divergence in sympatry and allopatry. Mol Ecol. 2017;26:5676–91. http://dx.doi.org/10.1111/mec.14276

5. Caprioli M, Ambrosini R, Boncoraglio G, Gatti E, Romano A, Romano M, et al. Clock gene variation is associated with breeding phenology and maybe under directional selection in the migratory barn swallow. PLoS One. 2012;7:e35140. http://dx.doi.org/10.1371/journal.pone.0035140

6. Saino N, Romano M, Caprioli M, Fasola M, Lardelli R, Micheloni P, et al. Timing of molt of barn swallows is delayed in a rare Clock genotype. PeerJ. 2013;1:e17. http://dx.doi.org/10.7717/peerj.17

7. Bazzi G, Ambrosini R, Caprioli M, Costanzo A, Liechti F, Gatti E, et al. Clock gene polymorphism and scheduling of migration: a geolocator study of the barn swallow Hirundo rustica. Sci Rep. 2015;5:12443. http://dx.doi.org/10.1038/srep12443

8. Saino N, Ambrosini R, Albetti B, Caprioli M, De Giorgio B, Gatti E, et al. Migration phenology and breeding success are predicted by methylation of a photoperiodic gene in the barn swallow. Sci Rep. 2017;7:45412. http://dx.doi.org/10.1038/srep45412

9. DLS announcement by Bionano Genomics at AGBT [Internet]. https://bionanogenomics.com/wp-content/uploads/2018/02/Bionano-AGBT2018-DLS_launch_final.pdf

10. Lewin HA, Robinson GE, Kress WJ, Baker WJ, Coddington J, Crandall KA, et al. Earth BioGenome Project: Sequencing life for the future of life. Proc Natl Acad Sci U S A. 2018;115:4325–33. http://dx.doi.org/10.1073/pnas.1720115115

11. Vertebrate Genomes Project [Internet]. [cited 2018 Oct 2]. Available from: https://vertebrategenomesproject.org

12. Vertebrate Genomes Project Phase 1 first data release [Internet]. Available from: https://vertebrategenomesproject.org/news/

13. Kadi F, Mouchiroud D, Sabeur G, Bernardi G. The compositional patterns of the avian genomes and their evolutionary implications. J Mol Evol. 1993;37:544–51. https://doi.org/10.1007/BF00160434

14. Koren S, Walenz BP, Berlin K, Miller JR, Bergman NH, Phillippy AM. Canu: scalable and accurate long-read assembly via adaptive k-mer weighting and repeat separation. Genome Res. 2017;27:722–36. http://dx.doi.org/10.1101/gr.215087.116

15. Korlach J, Gedman G, Kingan SB, Chin C-S, Howard JT, Audet J-N, et al. De novo PacBio long-read and phased avian genome assemblies correct and add to reference genes generated with intermediate and short reads. Gigascience. 2017;6:1–16. http://dx.doi.org/10.1093/gigascience/gix085

16. Andrews CB, Mackenzie SA, Gregory TR. Genome size and wing parameters in passerine birds. Proc Biol Sci. 2009;276:55–61. http://dx.doi.org/10.1098/rspb.2008.1012

17. Ellegren H. Evolutionary stasis: the stable chromosomes of birds. Trends Ecol Evol. 2010;25:283–91. http://dx.doi.org/10.1016/j.tree.2009.12.004

18. Morgulis A, Gertz EM, Schäffer AA, Agarwala R. WindowMasker: window-based masker for sequenced genomes. Bioinformatics. 2006;22:134–41. http://dx.doi.org/10.1093/bioinformatics/bti774

19. Smit AF, Hubley R, Green P. RepeatMasker Open-3.0. 1996–2010. http://www.repeatmasker.org

20. Stanke M, Steinkamp R, Waack S, Morgenstern B. AUGUSTUS: a web server for gene finding in eukaryotes. Nucleic Acids Res. 2004;32:W309–12. http://dx.doi.org/10.1093/nar/gkh379

21. Mistry J, Bateman A, Finn RD. Predicting active site residue annotations in the Pfam database. BMC Bioinformatics. 2007;8:298. http://dx.doi.org/10.1186/1471-2105-8-298

22. Altschul SF, Gish W, Miller W, Myers EW, Lipman DJ. Basic local alignment search tool. J Mol Biol. 1990;215:403–10. http://dx.doi.org/10.1016/S0022-2836(05)80360-2

23. Gallus gallus Proteins. NCBI. Available from: https://www.ncbi.nlm.nih.gov/genome/proteins/111?genome_assembly_id=374862

24. Simão FA, Waterhouse RM, Ioannidis P, Kriventseva EV, Zdobnov EM. BUSCO: assessing genome assembly and annotation completeness with single-copy orthologs. Bioinformatics [Internet]. 2015;31:3210–2. http://dx.doi.org/10.1093/bioinformatics/btv351

25. Zdobnov EM, Tegenfeldt F, Kuznetsov D, Waterhouse RM, Simão FA, Ioannidis P, et al. OrthoDB v9.1: cataloging evolutionary and functional annotations for animal, fungal, plant, archaeal, bacterial and viral orthologs. Nucleic Acids Res. 2017;45:D744–9. http://dx.doi.org/10.1093/nar/gkw1119

26. Gouy M, Guindon S, Gascuel O. SeaView version 4: A multiplatform graphical user interface for sequence alignment and phylogenetic tree building. Mol Biol Evol. 2010;27:221–4. http://dx.doi.org/10.1093/molbev/msp259

27. Castresana J. Selection of conserved blocks from multiple alignments for their use in phylogenetic analysis. Mol Biol Evol. 2000;17:540–52. http://dx.doi.org/10.1093/oxfordjournals.molbev.a026334

28. Guindon S, Dufayard J-F, Lefort V, Anisimova M, Hordijk W, Gascuel O. New algorithms and methods to estimate maximum-likelihood phylogenies: assessing the performance of PhyML 3.0. Syst Biol. 2010;59:307–21. http://dx.doi.org/10.1093/sysbio/syq010

29. Le SQ, Gascuel O. An improved general amino acid replacement matrix. Mol Biol Evol. 2008;25:1307–20. http://dx.doi.org/10.1093/molbev/msn067

30. Jarvis ED, Mirarab S, Aberer AJ, Li B, Houde P, Li C, et al. Whole-genome analyses resolve early branches in the tree of life of modern birds. Science. 2014;346:1320–31. http://dx.doi.org/10.1126/science.1253451

31. Jarvis ED, Mirarab S, Aberer AJ, Li B, Houde P, Li C, et al. Phylogenomic analyses data of the avian phylogenomics project. Gigascience. 2015;4:4. http://dx.doi.org/10.1186/s13742-014-0038-1

32. Selvatti AP, Gonzaga LP, Russo CA de M. A Paleogene origin for crown passerines and the diversification of the Oscines in the New World. Mol Phylogenet Evol. 2015;88:1–15. http://dx.doi.org/10.1016/j.ympev.2015.03.018

33. Claramunt S, Cracraft J. A new time tree reveals Earth history's imprint on the evolution of modern birds. Sci Adv. 2015;1:e1501005. http://dx.doi.org/10.1126/sciadv.1501005

34. Cabanettes F, Klopp C. D-GENIES: dot plot large genomes in an interactive, efficient and simple way. PeerJ. 2018;6:e4958. http://dx.doi.org/10.7717/peerj.4958

35. Völker M, Backström N, Skinner BM, Langley EJ, Bunzey SK, Ellegren H, et al. Copy number variation, chromosome rearrangement, and their association with recombination during avian evolution. Genome Res. 2010;20:503–11. http://dx.doi.org/10.1101/gr.103663.109

36. Pala I, Naurin S, Stervander M, Hasselquist D, Bensch S, Hansson B. Evidence of a neo-sex chromosome in birds. Heredity. 2012;108:264–72. http://dx.doi.org/10.1038/hdy.2011.70

37. Wirthlin M, Lovell PV, Jarvis ED, Mello CV. Comparative genomics reveals molecular features unique to the songbird lineage. BMC Genomics. 2014;15:1082. http://dx.doi.org/10.1186/1471-2164-15-1082

38. Kasai F, O'Brien PCM, Ferguson-Smith MA. Reassessment of genome size in turtle and crocodile based on chromosome measurement by flow karyotyping: close similarity to chicken. Biol Lett. 2012;8:631–5. http://dx.doi.org/10.1098/rsbl.2012.0141

39. Kawakami T, Smeds L, Backström N, Husby A, Qvarnström A, Mugal CF, et al. A high-density linkage map enables a second-generation collared flycatcher genome assembly and reveals the patterns of avian recombination rate variation and chromosomal evolution. Mol Ecol. 2014;23:4035–58. http://dx.doi.org/10.1111/mec.12810

40. Miller MM, Taylor RL Jr. Brief review of the chicken Major Histocompatibility Complex: the genes, their distribution on chromosome 16, and their contributions to disease resistance. Poult Sci. 2016;95:375–92. http://dx.doi.org/10.3382/ps/pev379

41. Smeds L, Warmuth V, Bolivar P, Uebbing S, Burri R, Suh A, et al. Evolutionary analysis of the female-specific avian W chromosome. Nat Commun. 2015;6:7330. http://dx.doi.org/10.1038/ncomms8330

42. Bellott DW, Skaletsky H, Cho T-J, Brown L, Locke D, Chen N, et al. Avian W and mammalian Y chromosomes convergently retained dosage-sensitive regulators. Nat Genet. 2017;49:387–94. http://dx.doi.org/10.1038/ng.3778

43. Itoh Y, Kampf K, Arnold AP. Molecular cloning of zebra finch W chromosome repetitive sequences: evolution of the avian W chromosome. Chromosoma. 2008;117:111–21. http://dx.doi.org/10.1007/s00412-007-0130-8

44. Komissarov AS, Galkina SA, Koshel EI, Kulak MM, Dyomin AG, O'Brien SJ, et al. New high copy tandem repeat in the content of the chicken W chromosome. Chromosoma. 2018;127:73–83. http://dx.doi.org/10.1007/s00412-017-0646-5

45. Genome 10K Community of Scientists. Genome 10K: a proposal to obtain whole-genome sequence for 10,000 vertebrate species. J Hered. 2009;100:659–74. http://dx.doi.org/10.1093/jhered/esp086

46. Zhang G, Li C, Li Q, Li B, Larkin DM, Lee C, et al. Comparative genomics reveals insights into avian genome evolution and adaptation. Science. 2014;346:1311–20. http://dx.doi.org/10.1126/science.1251385

47. Henson J, Tischler G, Ning Z. Next-generation sequencing and large genome assemblies. Pharmacogenomics. 2012;13:901–15. http://dx.doi.org/10.2217/pgs.12.72

48. Sela N, Kim E, Ast G. The role of transposable elements in the evolution of non-mammalian vertebrates and invertebrates. Genome Biol. 2010;11:R59. http://dx.doi.org/10.1186/gb-2010-11-6-

r59

49. Koepfli K-P, Paten B, Genome 10K Community of Scientists, O'Brien SJ. The Genome 10K Project: a way forward. Annu Rev Anim Biosci. 2015;3:57–111. http://dx.doi.org/10.1146/annurev-animal-090414-014900

50. Zhang G, Rahbek C, Graves GR, Lei F, Jarvis ED, Gilbert MTP. Genomics: Bird sequencing project takes off. Nature. 2015;522:34. http://dx.doi.org/10.1038/522034d

51. Pennisi E. Sequencing all life captivates biologists. Science. 2017;355:894–5. http://dx.doi.org/10.1126/science.355.6328.894

52. Teeling EC, Vernes SC, Dávalos LM, Ray DA, Gilbert MTP, Myers E, et al. Bat Biology, Genomes, and the Bat1K Project: To Generate Chromosome-Level Genomes for All Living Bat Species. Annu Rev Anim Biosci. 2018;6:23–46. http://dx.doi.org/10.1146/annurev-animal-022516-022811

53. Bleidorn C. Third generation sequencing: technology and its potential impact on evolutionary biodiversity research. System Biodivers. Taylor & Francis; 2016;14:1–8. https://doi.org/10.1080/14772000.2015.1099575

54. Hunt M, Newbold C, Berriman M, Otto TD. A comprehensive evaluation of assembly scaffolding tools. Genome Biol. 2014;15:R42. http://dx.doi.org/10.1186/gb-2014-15-3-r42

55. Burton JN, Adey A, Patwardhan RP, Qiu R, Kitzman JO, Shendure J. Chromosome-scale scaffolding of de novo genome assemblies based on chromatin interactions. Nat Biotechnol. 2013;31:1119–25. http://dx.doi.org/10.1038/nbt.2727

56. Lam ET, Hastie A, Lin C, Ehrlich D, Das SK, Austin MD, et al. Genome mapping on nanochannel arrays for structural variation analysis and sequence assembly. Nat Biotechnol. 2012;30:771–6. http://www.nature.com/articles/nbt.2303

57. Nowoshilow S, Schloissnig S, Fei J-F, Dahl A, Pang AWC, Pippel M, et al. The axolotl genome and the evolution of key tissue formation regulators. Nature. 2018;554:50–5. http://dx.doi.org/10.1038/nature25458

58. Gao Y, Wang H, Liu C, Chu H, Dai D, Song S, et al. De novo genome assembly of the red silk cotton tree (Bombax ceiba). Gigascience. 2018;7. http://dx.doi.org/10.1093/gigascience/giy051

Figure 1

Click here to access/download;Figure;Figure 1.tif

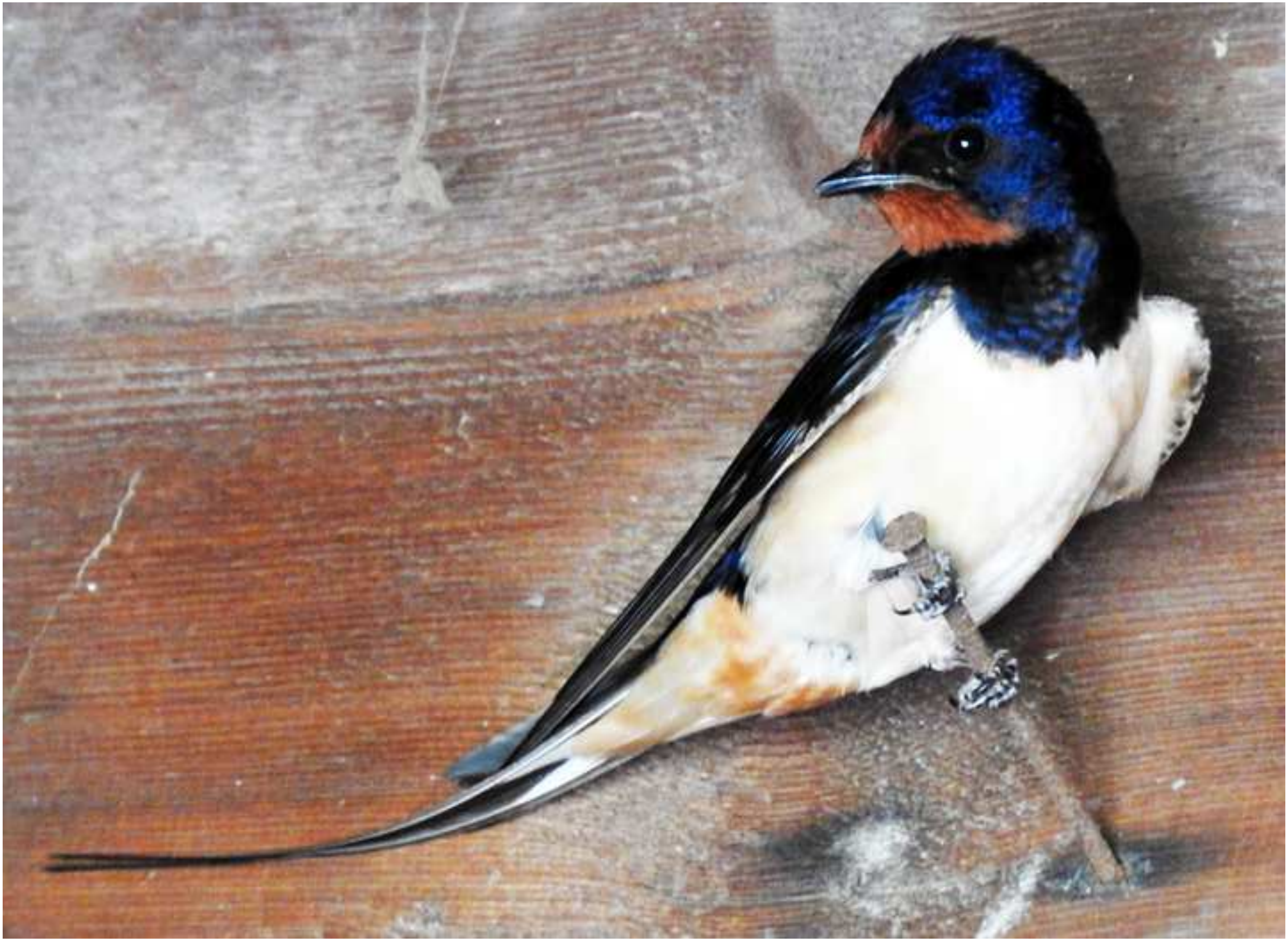

Figure 2

Click here to access/download;Figure;Figure 2.eps

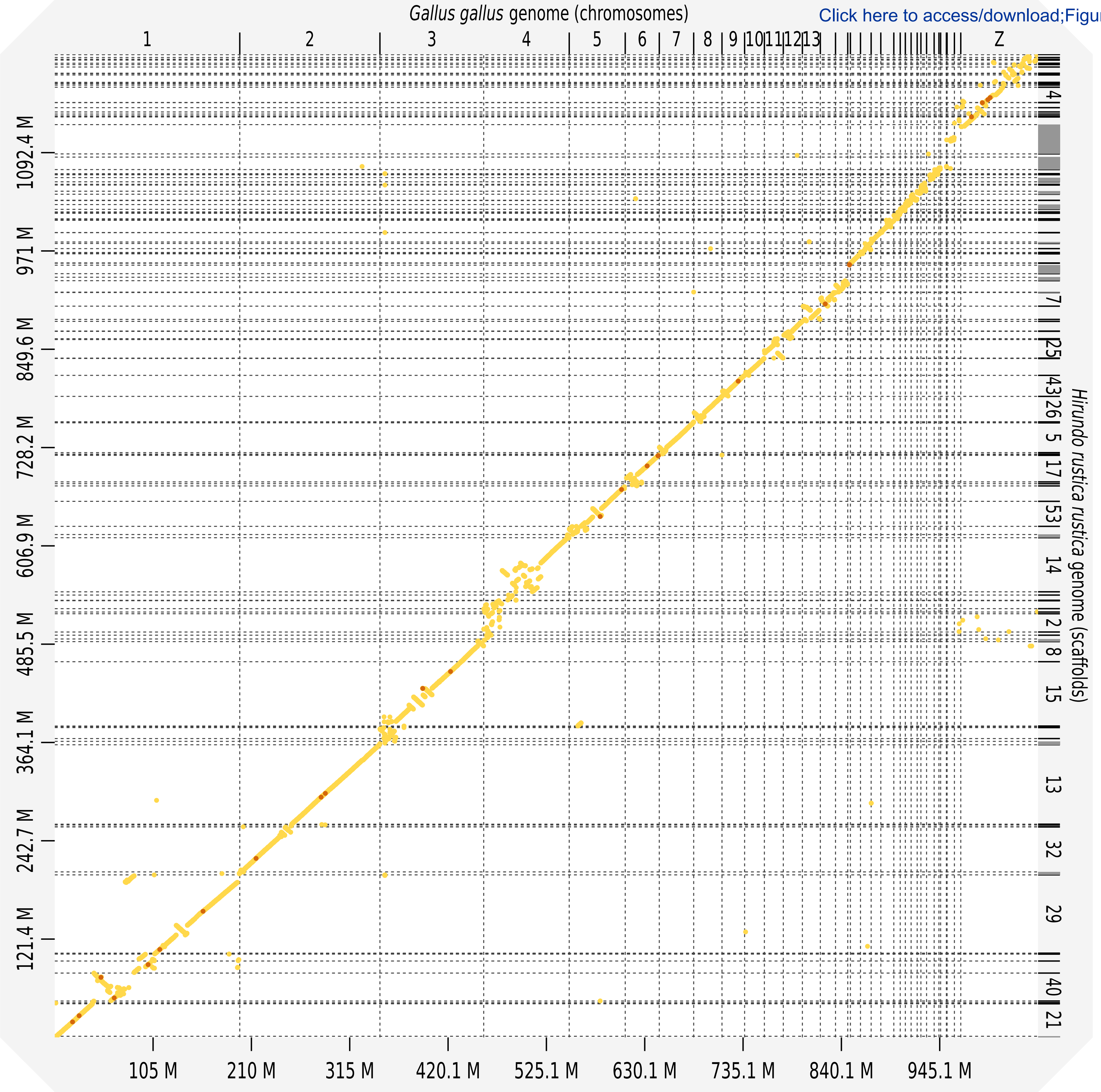

Supplementary Figure 1

Click here to access/download
Supplementary Material
Supplementary Figure 1.png

Supplementary Figure 2

Click here to access/download
Supplementary Material
Supplementary Figure 2.tif

Supplementary Figure 3

Click here to access/download
Supplementary Material
Supplementary Figure 3.png

Supplementary Figure 4

Click here to access/download
Supplementary Material
Supplementary Figure 4.png

Supplementary Figure 5

Click here to access/download
Supplementary Material
Supplementary Figure 5.tif

Supplementary Figure 6

Click here to access/download
Supplementary Material
Supplementary Figure 6.eps

Supplementary Table 1

Click here to access/download
Supplementary Material
Supplementary Table 1.xlsx
