# Supplementary material for: SMRT long reads and Direct Label and Stain optical maps allow the generation of a high-quality genome assembly for the European barn swallow (Hirundo rustica rustica)
Source: Gigascience. 2018 Nov 29;8(1):giy142. doi: 10.1093/gigascience/giy142 (PMC6324554; doi:10.1093/gigascience/giy142)
Supplement: response_to_reviewer_comments_original_submission.pdf [file giy142_response_to_reviewer_comments_original_submission.pdf]

Author's Response To Reviewer Comments

Close

Dear Editor,

First of all we would like to thank you and the reviewers for the prompt and detailed comments on our manuscript. We have addressed all the points raised, which resulted in improved clarity and appeal of the work.

Furthermore, during the initial review process, through communications with Erich Jarvis, the G10K Chair, regarding how to correctly acknowledge the G10K support of this work, we also understood that the Bionano DLS system has been utilized in several of their, as yet, unpublished genome assemblies. This fact, along with the correct acknowledgement to G10K approved by G10K Chair, has been inserted into the current version.

Below we provide detailed descriptions of the changes made as well as explanations of a couple of other unrequested modifications. The current version is slightly longer than the original submission, but we hope that you will agree that the extra length is justified by the content.

If you have any further queries regarding the submission, please do not hesitate to contact us.

Editor request:

Please ensure that there is sufficient methodological detail and we recommend that any relevant protocols are captured in protocols.io (there may be suitable protocols there for reuse or adapting, see our group collecting them https://www.protocols.io/groups/gigascience-journal). For validation we also recommend including a phylogenetic tree comparing where it fits some of the related sequenced species, especially in the light of the B10K project releasing so many bird genomes.

>We have now included an ML phylogenetic tree based on concatenated multiple gene alignments using our genome and those of some close relatives from the B10K project and other bird WGS projects. Our genome assembly places the barn swallow in the expected of the tree according to the literature (see text). Individual gene alignments have been added to the GigaDB submission associated with this work.

Reviewer reports:
Reviewer #1: ## General comments ##
The authors have produced a high-quality genome assembly of the European barn swallow, and important work that should allow the diverse research already done on the barn swallow to expand with a genomic basis.

The manuscript contains a lot of details about the methods, I would say much more than comparable manuscripts. This is very good, and very helpful for researchers interested in the methods the authors have used.

Be aware that the citations in the manuscript text link to a Paperpile reference, for instance this link: https://paperpile.com/c/R4nnD5/rZ3E. I cannot access the Google Document via that link. I guess this is the case for the bioRxiv version of this manuscript also. This will be removed in the processing of the manuscript before publication I guess, I just wanted you to be aware of it, because it can be a bit annoying.

>We removed any hyperlink from the document.

Specific comments

Abstract:
Line 9: This is a bit personal opinion, so you can ignore it, but I believe it is more accurate than what you have written: I prefer to refer to the digital version of a genome as a genome assembly. Genomes exist in individuals of a species, while genome assemblies exist in the computer. So you present a "high-quality genome assembly", and not a genome. You do this correct in line 32, but not necessary in the data description. I prefer "reference genome assembly", and not "reference genome", which would be something else.

>We modified the abstract according to the suggestion from the reviewer.

Data description:
Line 16: I find it strange that Molecular Ecology does not enforce release of this data (the Illumina based genome assembly). The editor of that paper should have held it back until the data was released. The scientific field does not progress as quickly as it could if people withhold data such as those guys do. It is great that you have released your data, as should be the default in all cases.

>We have once again formally requested the Journal to enforce the data release, explaining that are publishing a new HQ genome assembly for the barn swallow. We hope that this report will be taken into more serious consideration by the Journal.

Lines 77-80: You have a different font here for some reason.

>Solved.

Line 87: You have a comma instead of a punctuation mark in "5,2 Mbp".

>Solved.

Lines 173-176: Since you did this, I think you are correct in your conclusion, but the contigs might also just have not been placed. That is, the place the contigs (some of them at least) should have been was filled with a gap (Ns), and therefore the genomic location was in a way counted twice. First as the length of the contig, and then as the length of the gap. That could have explained some of the discrepancy, but since you found them to so similar to already assembled sequence, your explanation is better.

>We are unable to resolve with confidence haplotigs from potential gene duplication or indeed assembly errors for the time being. However, we draw the reviewer's attention to the last part of the results (lines 305-330), where we present specific arguments regarding

repeats associated with individual chromosomes.

Line 179: The difference between the estimate and your genome assembly could be the centromeres and the telomeres, unless these are captured well in your assembly. I guess they might be the "poorly assembled repeats" you mention.

>We added centromeric and telomeric regions as examples (lines 245-246). We also include a more developed discussion of assembly size and conservation with other bird genomes which we hope will address several comments that were congruent between reviewers.

"Annotation of genes and repeats"
I think you describe most of the other methods well, but I would have liked a bit more detail in this section. How was the assembly repeat-masked? Did you use the built in bird repetitive elements, or create a de novo library?

>We used Chicken repeat models from RepBase. This is now clarified in the text (lines 263-270).

Did you just run Augustus with Gallus gallus as species? That is, you did not train Augustus on your genome? Or did you use Gallus gallus genes in the form of proteins or transcripts mapped to the assembly to train Augustus?

>Yes, we used the Gallus gallus gene models in Augustus rather than training on the swallow genome where we did not have sufficient cDNA data to reliably train Augustus. In the longer term, improved annotation incorporating experimental data will be provided. We believe that the text is reasonably clear on this.

You could mention how you found the PFAM domains. Was it with HMMER or InterProScan or something else? You did not use the annotation for anything else than comparing to Gallus gallus?

>We used PFAMScan (which includes HMMER). This is now clarified in the text.

Lines 211-214: I don't understand your reasoning here. If 52 of the 54 avian genome sequences contain these 40 genes, then would not that suggest that the 2 genome assemblies that do not contain them, are incomplete? Or are these 2 phylogenetically separate from the other 52 in some way?

>In the end we removed this comment, different possibilities could explain the observation (systematic difficulty of sequencing particular genomic regions with short read technology, non-universal presence of genes etc…). This discussion is interesting, but probably adds little to, and is outside the scope of the current work.

Line 226: Moore's law does not say anything about microchip complexity, but it is about the number of transistors on a integrated circuit.

>We have removed the slightly cliched reference to Moore's law.

Line 262: I don't think you explicitly compare your scaffolds to the chromosomes. I guess most of the large chromosomes would be found in 2-3 scaffolds. Is this correct? The synteny

of birds is quite well conserved, so I think you could compare to chicken or zebra finch or something like that.

>We now provide a synthetic discussion of this issue as well as a table illustrating the numbers of scaffolds aligned to each chicken chromosome as well as the percentage of each chicken chromosome aligned (Table 2). We have also provided a slightly developed the discussion of sex chromosomes in the context of the overall genome size and the effect of long repeats such as centromeres and telomeres on our assembly.

Reviewer #2: This paper describes the sequencing and assembly of the European barn swallow using PacBio long reads and the latest technique for optical mapping. The result is a highly contiguous assembly that can benefit future genetic studies in barn swallows in particular and avian genomics in general.

The paper is well written and clear and I don't have any major concerns. Some general and specific comments follow below.

I was a bit surprised that the authors put so much focus on comparing their work with a previous Illumina-only based draft assembly (Safran et al 2016). It seems to me that the previous (not even publicly available) assembly was done very quick-and-dirty (using very few and short insert sizes, without any kind of curation at all) with the only purpose to facilitate SNP-calling with GBS data. Hence, it's not surprising at all that the new PacBio+Bionano assembly hundred-fold outperforms the previous draft, and I think the detailed comparison (both in text, table and supplementary table) is quite unnecessary, one single sentence mentioning this would have been enough.

>According to the reviewer's suggestion we have limited the emphasis to a single sentence mentioning the earlier genome assembly in the Context section of the manuscript (lines 82-83). In line with other specific comments from the reviewer with respect to this, we have also removed the comparisons with the earlier genome from tables.

The authors also mention that the old draft was based on a male and hence lack sequence from the W chromosome, but does not further comment on W scaffolds in their new assembly. Especially since they point out the difference, I think it's appropriate to elaborate on this a bit more. The W chromosome is included in the latest Gallus gallus assembly so I assume it should be included in figure 2, but it's very hard to distinguish the different microchromosomes in this figure. An improvement could be adding a zoom-in on the W as a separate figure, and/or in the text mention the number of scaffolds that align to Gallus W including their summed up length. Is it less, the same, or even more than the 7Mb found in chicken?

>We have provided a brief discussion of sex chromosomes in the context of the overall genome size and the effect of long repeats in regions such as centromeres and telomeres on our assembly (lines 323-330).

Specific comments:
page 2, line 77-79 wrong font type for parts of the text

>Same as Reviewer#1. Solved.

page 4, line 105: "were expected", should be "was expected"

>Corrected.

page 7, line 178: "The final assembly is slightly smaller than the previously estimated genome size (1.28 Gbp) [9], possibly reflecting an imprecise older estimate and/or the possibility that some poorly assembled repeats were discarded in the final step described above." I'm sure both these suggestions could be true, but isn't it even more likely that some parts of the genome actually can't be assembled, not even with PacBio? I'm thinking mostly of centromeres, and the W chromosome which (just as the Y chromosome) has turned out to be very hard to assemble. For example in the chicken, karyotype stains show that chromosome W is about half the size of the 82 Mb long chromosome Z, but only 7Mb could be assembled. Also the (although few) fragmented or missing BUSCO genes suggest that not the entire genome is assembled. So to me it would have been more surprising if the barn swallow assembly equaled the "real" genome size.

>This comment from the reviewer mirrors that of the other reviewer. Both were correct and we believe that we addressed them when we state “The final assembly is slightly smaller than the previously estimated genome size (1.28 Gbp) [16], possibly reflecting an imprecise older estimate, and/or the possibility that some repeated sequences (e.g. centromeric and telomeric low complexity regions) were either collapsed in the initial assembly steps or discarded in the final haplotig purging step described above.” Please also see responses to previous related points.

page 10, line 262: "[..] as the fully assembled karyotype would have an N50 of ~ 90 Mbp". Is this based on chicken, or is the karyotype of barn swallow known? Please add a reference.

>This refers to the chicken assembly, as now made clear in the text (line 373).

Supplementary figure 2. Add explanations for the abbreviations "RFU" and "LM". To someone not familiar with this type of analysis, the vertical blue numbers and the orange vertical lines are a bit unclear, maybe an explanation can be added to the figure text? Why are there two different ">200000"? I assume the right part of the figure (darker and lighter bands) shows the same as the plot - is there some point in showing both, and if so, maybe they can be separated into a) and b)?

>Supplementary Figure 2 represents the typical output of the Femtopulse instrument. As suggested by the reviewer we added details and explanations to the caption of the figure and separated it into a) and b).

Supplementary figure 4. Parts of the boxplots lie outside the figure. Y axis label "Length" should also include "(bp)". In Supplementary figure 3 (bottom panel) it seems like one read is almost 160kb long, but here (in Supp fig 4) the scale ends at 150kb - is there any particular reason for removing that outlier?

>We have corrected the figure according to the suggestions from the reviewer. The reason for the absence of 160 kbp long reads from the graph that the figure was produced with post-trimming reads as now indicated in the caption.

Supplementary figure 5. Please add labels to the axes also in the figure itself.

>We added the labels to the figure.

Supplementary table 1. This table contains so much information that it's a bit hard to grasp. Related to my first comment above, I don't think the comparison with the Safran assembly adds anything important here, and I recommend omitting the columns "Safran", "SMRT vs Safran" and "Final vs Safran" to make the table smaller. The new assembly is remarkable in itself and doesn't need this comparison to prove it.

>As already specified above, according to this and previous suggestion from the reviewer, we removed the comparison with the older assembly. We removed the comparison also from the main table in the text.

Also, I'm not sure I see the point in showing "Expected # of genes per contig"? It says that it's based on the average gene size based on chicken, but how is it actually calculated? Is the total gene count taken from chicken as well, or from the gene prediction? Does it at all take into account the uneven distribution of genes across the genome (mentioned in the main text, page 3 line 78-79)?

>The reviewer is completely correct in highlighting this issue. In fact the estimates were simply based on average gene length annotation in Chicken and did not take into account non-uniform gene density. This statistic was included to highlight the fact that most genes should be expected to be “completely” included within contigs (not necessarily the case for a short read assembly). However, given that in the current version of our manuscript we have greatly reduced the comparison to the previously reported Illumina WGS assembly, we therefore decided to omit these values from the current version.

Close
