# Supplementary material for: SMRT long reads and Direct Label and Stain optical maps allow the generation of a high-quality genome assembly for the European barn swallow (Hirundo rustica rustica)
Source: Gigascience. 2018 Nov 29;8(1):giy142. doi: 10.1093/gigascience/giy142 (PMC6324554; doi:10.1093/gigascience/giy142)
Supplement: reviewer_1_report_(original_submission) -- Ole K Tørresen — 8/24/2018 Reviewed [file giy142_reviewer_1_report_(original_submission).pdf]

Reviewer Report

Title: **Lilikoi: an R package for personalized pathway-based classification modeling using metabolomics data**

Version: **Original Submission** **Date:** 8/24/2018

Reviewer name: **Ole K Tørresen**

Reviewer Comments to Author:

General comments
The authors have produced a high-quality genome assembly of the European barn swallow, and important work that should allow the diverse research already done on the barn swallow to expand with a genomic basis.

The manuscript contains a lot of details about the methods, I would say much more than comparable manuscripts. This is very good, and very helpful for researchers interested in the methods the authors have used.

Be aware that the citations in the manuscript text link to a Paperpile reference, for instance this link: https://paperpile.com/c/R4nnD5/rZ3E. I cannot access the Google Document via that link. I guess this is the case for the bioRxiv version of this manuscript also. This will be removed in the processing of the manuscript before publication I guess, I just wanted you to be aware of it, because it can be a bit annoying.

Specific comments

Abstract:
Line 9: This is a bit personal opinion, so you can ignore it, but I believe it is more accurate than what you have written: I prefer to refer to the digital version of a genome as a genome assembly. Genomes exist in individuals of a species, while genome assemblies exist in the computer. So you present a "high-quality genome assembly", and not a genome. You do this correct in line 32, but not necessary in the data description. I prefer "reference genome assembly", and not "reference genome", which would be something else.

Data description:
Line 16: I find it strange that Molecular Ecology does not enforce release of this data (the Illumina based genome assembly). The editor of that paper should have held it back until the data was released. The scientific field does not progress as quickly as it could if people withhold data such as those guys do. It is great that you have released your data, as should be the default in all cases.

Lines 77-80: You have a different font here for some reason.

Line 87: You have a comma instead of a punctuation mark in "5,2 Mbp".

Lines 173-176: Since you did this, I think you are correct in your conclusion, but the contigs might also just have not been placed. That is, the place the contigs (some of them at least) should have been was filled with a gap (Ns), and therefore the genomic location was in a way counted twice. First as the length of the contig, and then as the length of the gap. That could have explained some of the discrepancy, but since you found them to so similar to already assembled sequence, your explanation is better.

Line 179: The difference between the estimate and your genome assembly could be the centromeres and

the telomeres, unless these are captured well in your assembly. I guess they might be the "poorly assembled repeats" you mention.

"Annotation of genes and repeats"
I think you describe most of the other methods well, but I would have liked a bit more detail in this section. How was the assembly repeat-masked? Did you use the built in bird repetitive elements, or create a de novo library? Did you just run Augustus with Gallus gallus as species? That is, you did not train Augustus on your genome? Or did you use Gallus gallus genes in the form of proteins or transcripts mapped to the assembly to train Augustus? You could mention how you found the PFAM domains. Was it with HMMER or InterProScan or something else? You did not use the annotation for anything else than comparing to Gallus gallus?

Lines 211-214: I don't understand your reasoning here. If 52 of the 54 avain genome sequences contain these 40 genes, then would not that suggest that the 2 genome assemblies that do not contain them, are incomplete? Or are these 2 phylogenetically separate from the other 52 in some way?

Line 226: Moore's law does not say anything about microchip complexity, but it is about the number of transistors on a integrated circuit.

Line 262: I don't think you explicitly compare your scaffolds to the chromosomes. I guess most of the large chromosomes would be found in 2-3 scaffolds. Is this correct? The synteny of birds is quite well conserved, so I think you could compare to chicken or zebra finch or something like that.

Level of Interest

Please indicate how interesting you found the manuscript: Choose an item.

Quality of Written English

Please indicate the quality of language in the manuscript: Choose an item.

Declaration of Competing Interests

Please complete a declaration of competing interests, considering the following questions:

- Have you in the past five years received reimbursements, fees, funding, or salary from an organisation that may in any way gain or lose financially from the publication of this manuscript, either now or in the future?
- Do you hold any stocks or shares in an organisation that may in any way gain or lose financially from the publication of this manuscript, either now or in the future?
- Do you hold or are you currently applying for any patents relating to the content of the manuscript?
- Have you received reimbursements, fees, funding, or salary from an organization that holds or has applied for patents relating to the content of the manuscript?
- Do you have any other financial competing interests?
- Do you have any non-financial competing interests in relation to this paper?

If you can answer no to all of the above, write 'I declare that I have no competing interests' below. If your reply is yes to any, please give details below.

I declare that I have no competing interests

I agree to the open peer review policy of the journal. I understand that my name will be included on my report to the authors and, if the manuscript is accepted for publication, my named report including any attachments I upload will be posted on the website along with the authors' responses. I agree for my report to be made available under an Open Access Creative Commons CC-BY license (http://creativecommons.org/licenses/by/4.0/). I understand that any comments which I do not wish to be included in my named report can be included as confidential comments to the editors, which will not be published.

I agree to the open peer review policy of the journal

To further support our reviewers, we have joined with Publons, where you can gain additional credit to further highlight your hard work (see: https://publons.com/journal/530/gigascience). On publication of this paper, your review will be automatically added to Publons, you can then choose whether or not to claim your Publons credit. I understand this statement. Yes
