# Supplementary material for: SMRT long reads and Direct Label and Stain optical maps allow the generation of a high-quality genome assembly for the European barn swallow (Hirundo rustica rustica)
Source: Gigascience. 2018 Nov 29;8(1):giy142. doi: 10.1093/gigascience/giy142 (PMC6324554; doi:10.1093/gigascience/giy142)
Supplement: reviewer_2_report_(original_submission) -- Linnea Smeds — 9/12/2018 Reviewed [file giy142_reviewer_2_report_(original_submission).pdf]

Reviewer Report

Title: **Lilikoi: an R package for personalized pathway-based classification modeling using metabolomics data**

Version: **Original Submission** **Date:** 9/12/2018

Reviewer name: **Linnea Smeds**

Reviewer Comments to Author:

This paper describes the sequencing and assembly of the European barn swallow using PacBio long reads and the latest technique for optical mapping. The result is a highly contiguous assembly that can benefit future genetic studies in barn swallows in particular and avian genomics in general.

The paper is well written and clear and I don't have any major concerns. Some general and specific comments follow below.

I was a bit surprised that the authors put so much focus on comparing their work with a previous Illumina-only based draft assembly (Safran et al 2016). It seems to me that the previous (not even publicly available) assembly was done very quick-and-dirty (using very few and short insert sizes, without any kind of curation at all) with the only purpose to facilitate SNP-calling with GBS data. Hence, it's not surprising at all that the new PacBio+Bionano assembly hundred-fold outperforms the previous draft, and I think the detailed comparison (both in text, table and supplementary table) is quite unnecessary, one single sentence mentioning this would have been enough.

The authors also mention that the old draft was based on a male and hence lack sequence from the W chromosome, but does not further comment on W scaffolds in their new assembly. Especially since they point out the difference, I think it's appropriate to elaborate on this a bit more. The W chromosome is included in the latest Gallus gallus assembly so I assume it should be included in figure 2, but it's very hard to distinguish the different microchromosomes in this figure. An improvement could be adding a zoom-in on the W as a separate figure, and/or in the text mention the number of scaffolds that align to Gallus W including their summed up length. Is it less, the same, or even more than the 7Mb found in chicken?

Specific comments:
page 2, line 77-79 wrong font type for parts of the text

page 4, line 105: "were expected", should be "was expected"

page 7, line 178: "The final assembly is slightly smaller than the previously estimated genome size (1.28 Gbp) [9], possibly reflecting an imprecise older estimate and/or the possibility that some poorly assembled repeats were discarded in the final step described above." I'm sure both these suggestions could be true, but isn't it even more likely that some parts of the genome actually can't be assembled, not even with PacBio? I'm thinking mostly of centromeres, and the W chromosome which (just as the Y chromosome) has turned out to be very hard to assemble. For example in the chicken, karyotype stains show that chromosome W is about half the size of the 82 Mb long chromosome Z, but only 7Mb could be assembled. Also the (although few) fragmented or missing BUSCO genes suggest that not the entire genome is assembled. So to me it would have been more surprising if the barn swallow assembly equaled the "real" genome size.

page 10, line 262: "[..] as the fully assembled karyotype would have an N50 of ~ 90 Mbp". Is this based on chicken, or is the karyotype of barn swallow known? Please add a reference.

Supplementary figure 2. Add explanations for the abbreviations "RFU" and "LM". To someone not familiar with this type of analysis, the vertical blue numbers and the orange vertical lines are a bit unclear, maybe an explanation can be added to the figure text? Why are there two different ">200000"? I assume the right part of the figure (darker and lighter bands) shows the same as the plot - is there some point in showing both, and if so, maybe they can be separated into a) and b)?

Supplementary figure 4. Parts of the boxplots lie outside the figure. Y axis label "Length" should also include "(bp)". In Supplementary figure 3 (bottom panel) it seems like one read is almost 160kb long, but here (in Supp fig 4) the scale ends at 150kb - is there any particular reason for removing that outlier?

Supplementary figure 5. Please add labels to the axes also in the figure itself.

Supplementary table 1. This table contains so much information that it's a bit hard to grasp. Related to my first comment above, I don't think the comparison with the Safran assembly adds anything important here, and I recommend omitting the columns "Safran", "SMRT vs Safran" and "Final vs Safran" to make the table smaller. The new assembly is remarkable in itself and doesn't need this comparison to prove it.
Also, I'm not sure I see the point in showing "Expected # of genes per contig"? It says that it's based on the average gene size based on chicken, but how is it actually calculated? Is the total gene count taken from chicken as well, or from the gene prediction? Does it at all take into account the uneven distribution of genes across the genome (mentioned in the main text, page 3 line 78-79)?

Level of Interest

Please indicate how interesting you found the manuscript: Choose an item.

Quality of Written English

Please indicate the quality of language in the manuscript: Choose an item.

Declaration of Competing Interests

Please complete a declaration of competing interests, considering the following questions:

- Have you in the past five years received reimbursements, fees, funding, or salary from an organisation that may in any way gain or lose financially from the publication of this manuscript, either now or in the future?
- Do you hold any stocks or shares in an organisation that may in any way gain or lose financially from the publication of this manuscript, either now or in the future?
- Do you hold or are you currently applying for any patents relating to the content of the manuscript?
- Have you received reimbursements, fees, funding, or salary from an organization that holds or has applied for patents relating to the content of the manuscript?
- Do you have any other financial competing interests?
- Do you have any non-financial competing interests in relation to this paper?

If you can answer no to all of the above, write 'I declare that I have no competing interests' below. If your reply is yes to any, please give details below.

I declare that I have no competing interests

I agree to the open peer review policy of the journal. I understand that my name will be included on my report to the authors and, if the manuscript is accepted for publication, my named report including any attachments I upload will be posted on the website along with the authors' responses. I agree for my report to be made available under an Open Access Creative Commons CC-BY license (http://creativecommons.org/licenses/by/4.0/). I understand that any comments which I do not wish to be included in my named report can be included as confidential comments to the editors, which will not be published.

I agree to the open peer review policy of the journal

To further support our reviewers, we have joined with Publons, where you can gain additional credit to further highlight your hard work (see: https://publons.com/journal/530/gigascience). On publication of this paper, your review will be automatically added to Publons, you can then choose whether or not to claim your Publons credit. I understand this statement. Yes
